# TRBC2-targeting antibody–drug conjugates for the treatment of T cell cancers

**DOI:** 10.1038/s43018-025-01069-z

**Published:** 2025-12-22

**Authors:** Jiaxin Ge, Joshua Urban, Sarah R. DiNapoli, Bum Seok Lee, Taha Ahmedna, Tushar D. Nichakawade, Brian J. Mog, Steve Lu, Xuyang Li, Nikita Marcou, Stephanie Glavaris, Jacqueline Douglass, Jin Liu, Maximilian F. Konig, Evangeline Watson, Maria Popoli, J. David Peske, Sima Rozati, Cole H. Sterling, Nina Wagner-Johnston, Richard F. Ambinder, Kathy Gabrielson, Charles G. Mullighan, Nickolas Papadopoulos, Chetan Bettegowda, Drew M. Pardoll, Shibin Zhou, Surojit Sur, Kenneth W. Kinzler, Bert Vogelstein, Suman Paul

**Affiliations:** 1 Ludwig Center and Lustgarten Laboratory, Sidney Kimmel Comprehensive Cancer Center, Johns Hopkins University School of Medicine, Baltimore, MD, USA.; 2 Howard Hughes Medical Institute, Chevy Chase, MD, USA.; 3 Department of Chemical and Biomolecular Engineering, Johns Hopkins University, Baltimore, MD, USA.; 4 Institute for NanoBioTechnology, Johns Hopkins University, Baltimore, MD, USA.; 5 Department of Biomedical Engineering, Johns Hopkins University, Baltimore, MD, USA.; 6 Department of Oncology, Johns Hopkins School of Medicine, Baltimore, MD, USA.; 7 Division of Rheumatology, Department of Medicine, Johns Hopkins University School of Medicine, Baltimore, MD, USA.; 8 Department of Pathology, Johns Hopkins School of Medicine, Baltimore, MD, USA.; 9 Department of Dermatology, Johns Hopkins School of Medicine, Baltimore, MD, USA.; 10 Division of Hematologic Malignancies and Bone Marrow Transplantation, Department of Oncology, Johns Hopkins School of Medicine, Baltimore, MD, USA.; 11 Department of Pathology, St. Jude Children’s Research Hospital, Memphis, TN, USA.; 12 Department of Neurosurgery, Johns Hopkins University School of Medicine, Baltimore, MD, USA.; 13 Bloomberg–Kimmel Institute for Cancer Immunotherapy, Sidney Kimmel Comprehensive Cancer Center, Baltimore, MD, USA.; 14 Present address: Division of Hematologic Malignancies and Bone Marrow Transplantation, Department of Oncology, Johns Hopkins School of Medicine, Baltimore, MD, USA.

## Abstract

Antibody–drug conjugates (ADCs) have been remarkably successful in treating solid and hematological malignancies. Generation of ADCs for T cell cancers is challenging because the ADCs must selectively target cancerous T cells while sparing some normal T cells necessary for immune function. T cells express one of two *TRBC* alleles: *TRBC1* or *TRBC2*. Normal T cells are composed of about 40% *TRBC1*-expressing and 60% *TRBC2*-expressing cells. In contrast, T cell malignancies are characterized by the clonal expression of either *TRBC1* or *TRBC2*. Selective targeting of *TRBC1* or *TRBC2* enables the killing of cancer cells but preserves about 60–40% of the normal T cells. To enable such a therapy for cancers expressing *TRBC2*, here we developed a high-affinity anti-TRBC2 antibody. An ADC generated with this antibody and a pyrrolobenzodiazepine dimer payload showed specific killing of TRBC2^+^ cancers in vitro and in mouse models. The anti-TRBC2 ADC provides a promising, off-the-shelf therapy for patients with T cell cancers.

About 100,000 patients each year are diagnosed with T cell leukemias or lymphomas, collectively known as T cell cancers^1,2^. Patients with relapsed T cell cancers have limited therapeutic options and an estimated 5-year survival rate of 7–38% (refs. 3,4). In comparison, patients with B cell cancers now have access to innovative antibody and chimeric antigen receptor (CAR), T cell-based therapies that target pan-B cell antigens, leading to improved survival^5,6^. The development of similar therapeutics targeting T cell cancers has been difficult. Cancerous and normal B and T cells display similar antigens on their cell surface. Targeting pan-B cell antigens is viable because the resulting depletion of normal B cells is generally well tolerated^5–7^. However, a similar targeting of pan-T cell antigens often leads to profound immunosuppression, resulting in severe and sometimes fatal infections^8,9^. Thus, T cell cancers necessitate a more precise targeting of malignant T cells and preservation of some healthy T cells. Most normal and neoplastic T cells express the αβ T cell receptor (TCR) on their cell surface. Among T cell neoplasms, most (>90%) cutaneous T cell lymphomas (CTCLs) and peripheral T cell lymphomas express the αβ TCR^10^. In addition, a substantial portion (30–50%) of T cell acute lymphoblastic leukemias (T-ALLs) expresses the αβ TCR^11^. Each T cell features a distinct αβ TCR, which is generated through the somatic recombination of variable (*TRBV*), diversity (*TRBD*), joining (*TRBJ*) and constant (*TRBC*) gene segments. T cells select one of two available *TRBC* alleles: *TRBC1* or *TRBC2* (ref. 12) and normal αβ T cells consist of about ~40% TRBC1^+^ and ~60% TRBC2^+^ cells^13,14^. In contrast, as clonal outgrowth of a specific neoplastic T cell ultimately leads to T cell leukemias and lymphomas, all neoplastic T cells carry the same distinct TCR sequence. Thus, the cancerous T cells in a given individual are exclusively either TRBC1^+^ or TRBC2^+^ (refs. 13,14). Consequently, targeting the cancer cell-specific TRBC segment spares about 60–40% of the normal T cells, which may partially retain cellular immunity.

Recent work has taken advantage of this clonal TRBC expression in T cell cancers with the generation of TRBC1-targeting CAR T cells and ADCs, which specifically deplete the TRBC1^+^ cancerous and normal T cells while preserving the TRBC2^+^ normal T cells^13,15^. TRBC1-targeting therapies have demonstrated efficacy in pre-clinical studies^13,15^ and in a subset of patients with TRBC1^+^ T cell lymphomas in a phase 1/2 clinical trial^16^. However, a TRBC2^+^-targeting antibody with sufficient affinity and specificity to serve as an ADC is unavailable and is required to treat patients with TRBC2^+^ T cell cancers.

In this study, we used phage display to generate a high-affinity anti-TRBC2 antibody. The antibody distinctly identified T cells harboring TRBC2^+^ TCRs and lacks binding to TRBC1^+^ TCRs. We demonstrated that the anti-TRBC2 antibody was rapidly endocytosed on TCR binding, thereby allowing the delivery of cytotoxic payloads into the cancer cells. Finally, we showed that an ADC generated using the anti-TRBC2 antibody specifically killed TRBC2^+^ cancer cell lines and patient-derived cancers and induced tumor regression in xenogeneic mouse models of T cell cancers.

## Results

### Anti-TRBC2 antibody generation

**Extended Data Fig. 1 |:**
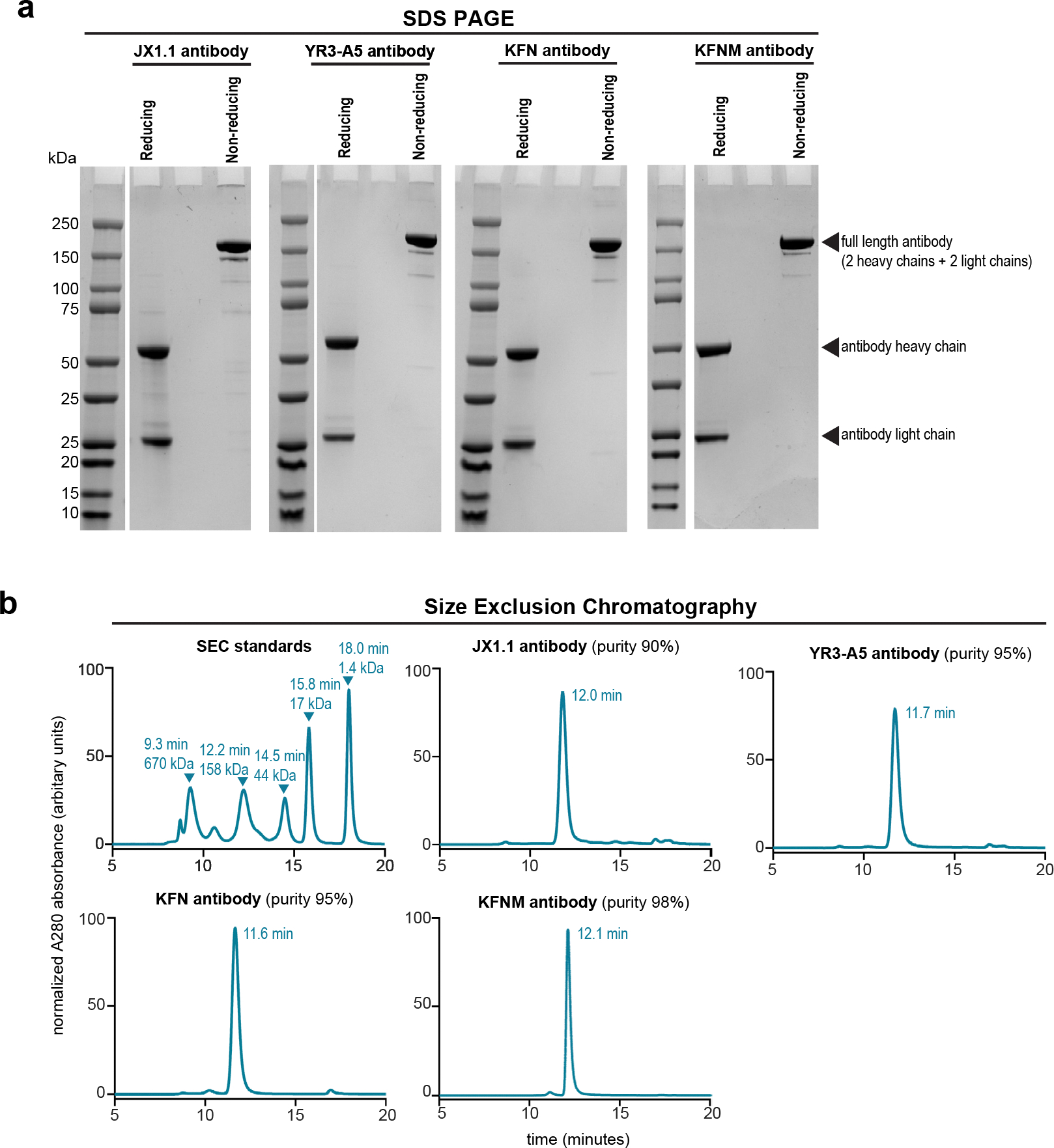
Anti-TRBC2 antibody production. **a**, SDS PAGE and Coomassie Blue stain of the indicated anti-TRBC2 antibodies under reducing and non-reducing conditions. Number of independent repeats = 1 **b**, Size exclusion chromatography (SEC) of the SEC standards and the indicated anti-TRBC2 antibodies. Data from one experiment.

**Extended Data Fig. 2 |:**
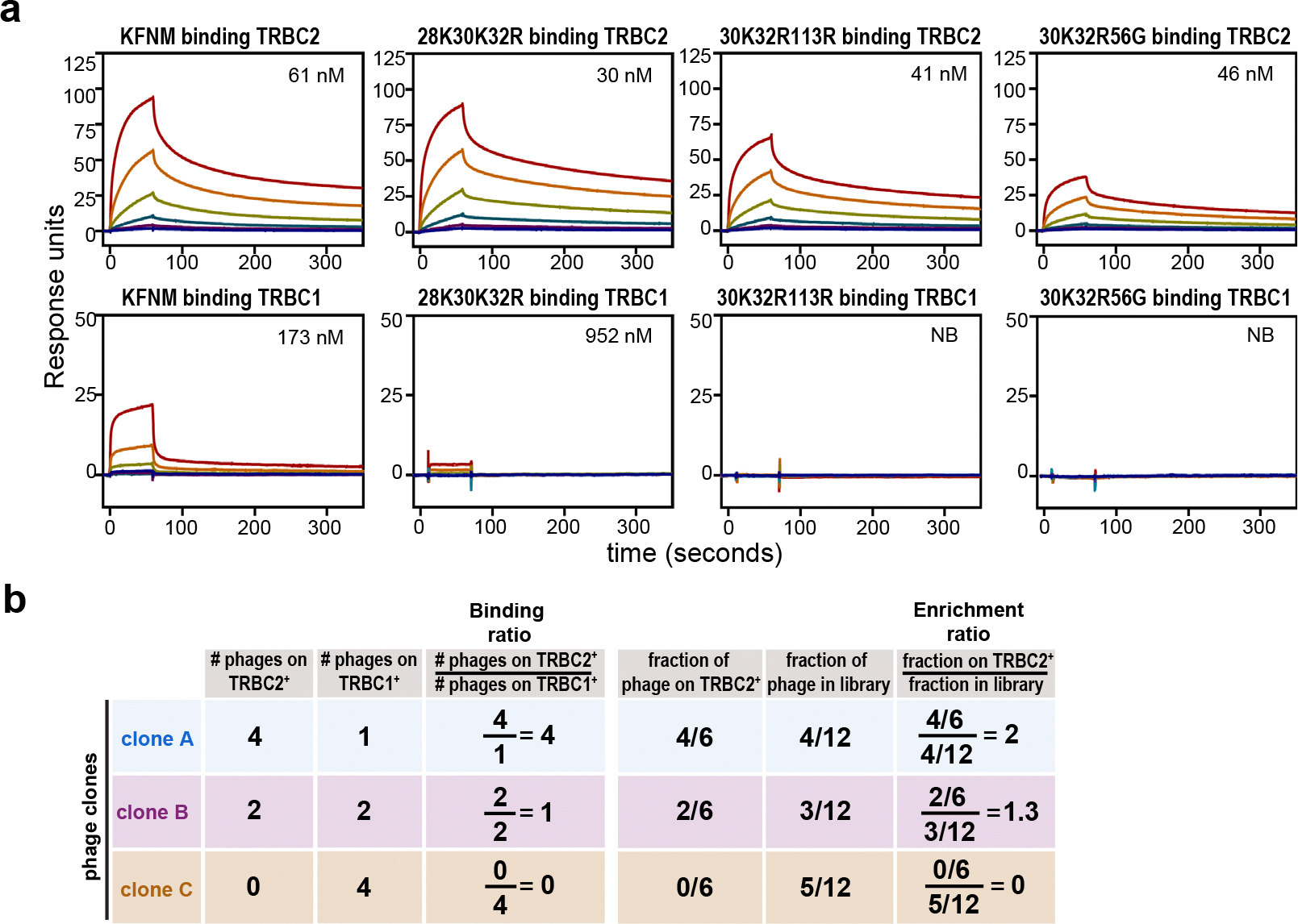
SPR measurements and explanation of SLISY ratios. **a**, Antibody binding to TRBC1 and TRBC2 proteins was measured with multiple-cycle kinetics by SPR. Antibodies were captured on CM5 chips, followed by injection of TRBC2 or TRBC1 proteins at the indicated concentrations. The affinities of each antibody to TRBC1 and TRBC2 proteins are indicated on the graph. NB = no binding. Data representative of one experiment. **b**, Example of calculation of binding ratio and enrichment ratio of the phage display illustrated in Fig. 1b.

**Fig. 1 |:**
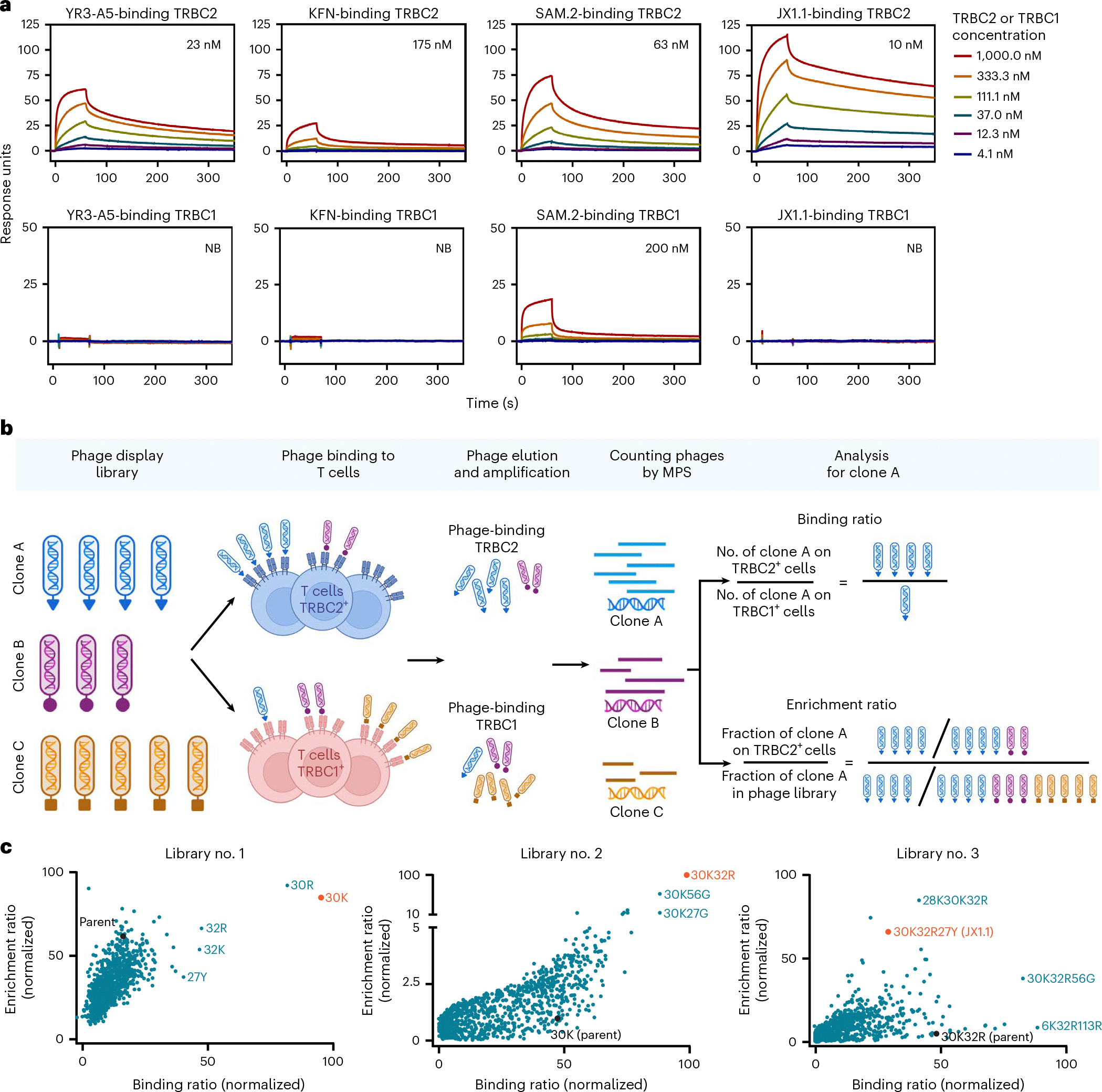
TRBC2-specific antibody generation by phage display. **a**, Antibody binding to TRBC1 (bottom) and TRBC2 (top) measured using multiple-cycle kinetics by SPR. Antibodies were captured on CM5 chips followed by injection of TRBC2 or TRBC1 proteins at the indicated concentrations. The affinities of each antibody to TRBC1 and TRBC2 proteins are indicated on the graph. **b**, Illustration showing the steps of phage display. The phage library was applied to T cell lines expressing TRBC2 or TRBC1. The phages were eluted, amplified in *Escherichia coli*, followed by MPS of the six CDRs to count the number of unique phage clones bound to TRBC2-expressing or TRBC1-expressing cells. The binding ratio and enrichment ratio of each phage clone were calculated as shown in the figure. **c**, Binding ratio versus enrichment ratio for all clones on SLISY. In library no. 1, the YR3-A5 phage (parent) is shown in black. The phages with high binding or enrichment ratios are identified by their point mutations, with the top clone in each library shown in orange. The ratios were normalized for distribution on a scale of 0–100 to enable graphic depiction. Data in **a** represent two (YR3-A5) or three (KFN, SAM.2 and JX1.1) independent experiments; data in **c** are from one experiment. Icons in **b** created using BioRender.com. NB, no binding.

Three groups recently described the generation of anti-TRBC2 antibodies: the KFN antibody^17^, the SAM.2 antibody^18,19^ and the YR3-A5 antibody^20^. We expressed recombinant YR3-A5, KFN and KFNM (a variant of KFN reported to have improved binding to TRBC2 (ref. 21)) antibodies by transient transfection of Chinese hamster ovary (CHO) cells. Sodium dodecylsulfate–polyacrylamide gel electrophoresis and size exclusion chromatography (SEC) demonstrated the production of ≥90% pure anti-TRBC2 antibodies at the expected molecular masses (Extended Data Fig. 1a,b). We then characterized each antibody’s affinity for the TRBC1 and TRBC2 proteins by surface plasmon resonance (SPR). YR3-A5 and KFN antibodies bound the TRBC2 protein with a dissociation constant (*K*_D_) of 23 nM and 175 nM, respectively (Fig. 1a and Supplementary Table 1). Neither antibody showed binding to the TRBC1 protein (Fig. 1a and Supplementary Table 1). Although the KFNM antibody showed increased affinity to the TRBC2 protein (*K*_D_, 61 nM) when compared to KFN, the KFNM antibody also retained binding to the TRBC1 protein (*K*_D_, 173 nM) (Extended Data Fig. 2a and Supplementary Table 1). Similarly, we characterized the commercially available SAM.2 antibody and found that it binds both the TRBC2 (*K*_D_, 63 nM) and the TRBC1 proteins (*K*_D_, 200 nM) (Fig. 1a and Supplementary Table 1). The therapeutic antibodies targeting B or T cells in clinical use bind with high affinity (low *K*_D_, ≤10 nM)^22–29^ to their target antigens and lack binding to unrelated antigens. Thus, the available anti-TRBC2 antibodies (YR3-A5, KFN, KFNM and SAM.2) did not appear to be optimally suited for development as a therapeutic anti-TRBC2 antibody.

To generate an anti-TRBC2 antibody with a higher binding affinity to TRBC2 and limited binding to TRBC1, we modified the sequence of the YR3-A5, single-chain, variable fragment (scFv). We substituted each amino acid residue of YR3-A5 for the other 19 canonical amino acids in the 6 complementarity-determining regions (CDRs), including the heavy-chain CDRs (CDR-H1 to CDR-H3) and the light-chain CDRs (CDR-L1 to CDR-L3), generating 1,406 unique antibody clones (Methods). The resultant scFv library was cloned into a phagemid vector. The scFv-expressing phages were expanded and applied to isogenic TRBC2^+^ cells (wild-type HPB-ALL cells expressing TRBC2^+^ TCR) or TRBC1^+^ cells (HPB-ALL cells CRISPR edited to express TRBC1^+^ TCR as previously described^15^) (Fig. 1b). We then used a massively parallel sequencing (MPS)-based assay called sequencing-linked immunosorbent assay (SLISY)^30^ to evaluate these clones. The scFv-expressing phages were sorted based on the binding ratio (ratio of the number of phages of a given clone binding TRBC2^+^ cells to those binding TRBC1^+^ cells) as well as enrichment ratio (ratio of the fraction of a clone among all clones that bound TRBC2^+^ cells to the fraction of the same phage clones among all clones in the original phage library) (Fig. 1b and Extended Data Fig. 2b). The scFv-bearing phages with selective and high affinity binding to TRBC2^+^ cells should ideally demonstrate both high binding and enrichment ratios. As expected, the vast majority of amino acid substitutions led to reduced binding or enrichment ratios (Fig. 1c), signifying loss of affinity or specificity for TRBC2. However, a few phage clones with substitutions in CDR-L1 positions 30 (V30K and V30R) and 32 (S32R and S32K) and CDR-H1 position 27 (R27Y) (Fig. 1c, library no. 1) demonstrated high binding or enrichment ratios.

We then constructed a second phagemid library using the CDR-L1 Val30Lys as the backbone and substituted each amino acid in all 6 CDRs (except amino acid 30Lys) with the other 19 amino acids. Using this second library and SLISY, we identified scFv-expressing phages with mutations in CDR-L1 position 32 (V30K, S32R) and CDR-H1 positions 27 (V30K, R27Y) and 56 (V30K, N56Q) (Fig. 1c, library no. 2) with binding and enrichment ratios above those of the V30K parental clone. We iterated this process, constructing a third phagemid library using CDR-L1 V30K, S32R as the backbone, and substituted each amino acid in all CDRs (except CDR-L1 amino acids 30K and 32R) with the other 19 amino acids. Evaluation of this third library with SLISY revealed several phage clones with higher binding or enrichment ratios ((N28K, V30K, S32R), (V30K, S32R, R27Y), (V30K, S32R, N56G) and (V30K, S32R, F113R)) (Fig. 1c, library no. 3).

Finally, we generated recombinant anti-TRBC2 antibodies that incorporated the top scFv sequences from library no. 3 (Extended Data Fig. 1a,b). SPR using recombinant TRBC2 protein revealed that clone V30K, S32R, R27Y (henceforth JX1.1) bound with the highest affinity (*K*_D_, 10 nM) among all the tested clones (Fig. 1a, Extended Data Fig. 2a and Supplementary Table 1). The high affinity was mediated by the slower off-rate (*k*_d_, 0.0013 s^−1^; Supplementary Table 1). A slower off-rate is advantageous because it increases the probability of antibody internalization, aiding ADC uptake and target cell killing^31^. Moreover, JX1.1 did not appreciably bind to recombinant TRBC1 protein (Fig. 1a and Supplementary Table 1). Given that the affinity of JX1.1 for TRBC2 was comparable to that of therapeutic antibodies used in the clinic and that it did not demonstrate binding to TRBC1, we focused on JX1.1 for further development.

### Anti-TRBC2 antibody specifically binds to TRBC2^+^ cells

**Fig. 2 |:**
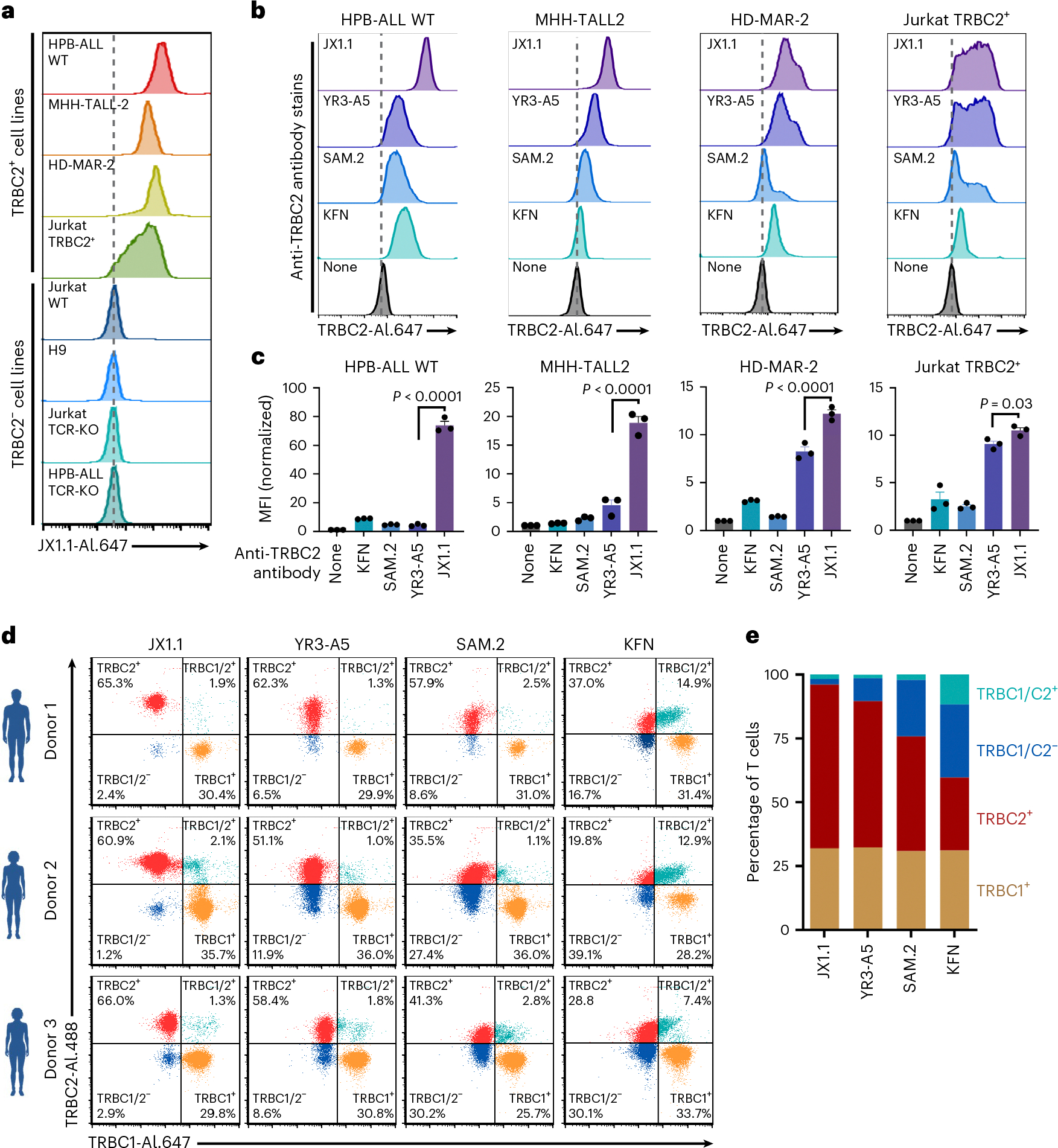
Anti-TRBC2 antibody shows specific binding to TRBC2^+^ cells. **a**, Flow cytometry histogram depicting binding of JX1.1 anti-TRBC2 antibody to the indicated T cell cancer cell lines. The data represent one experiment. **b**,**c**, Flow cytometry histogram depicting binding of the indicated anti-TRBC2 antibody clones to the four TRBC2-expressing T cell lines (**b**), with aggregate mean fluorescence intensity (MFI) from three experiments plotted (**c**). In **a** and **c**, cells were stained with the indicated anti-TRBC2 primary antibodies harboring a mouse immunoglobulin (Ig)G2a crystallizable fragment (Fc), followed by secondary staining with an anti-mouse IgG–Alexa Fluor-647 antibody. In ‘no primary antibody’ cells were stained with only the anti-mouse IgG–Alexa Fluor-647 antibody. The bar graphs represent mean ± s.e.m. *P* values were obtained by one-way analysis of variance (ANOVA) with Šídák’s multiple-comparison test. **d**,**e**, Flow cytometry of normal human T cells isolated from three different donors, followed by staining with the indicated anti-TRBC2 antibodies in combination with anti-TRBC1 antibody (clone JOVI.1) (**d**). Plots of the mean percentage of total T cells, identified as only TRBC1^+^, TRBC2^+^, TRBC1/2^+^ or TRBC1/2^−^ (**e**). In **d**, the cells were stained with the indicated primary anti-TRBC2 antibodies with a mouse IgG2a Fc, followed by secondary staining with an anti-mouse IgG2a–Alexa Fluor-488 antibody. For TRBC1 stain, cells were stained with the primary anti-TRBC1 antibody (clone JOVI.1) with a human Fc and a carboxyterminal 6× histidine tag, and were detected using secondary staining with an anti-His Alexa Fluor-647 antibody.

Flow cytometry demonstrated that the JX1.1 anti-TRBC2 antibody specifically bound to T cell lines with endogenous TRBC2^+^ expression (wild-type HPB-ALL, MHH-TALL2 and HD-MAR-2)^13,14^ and a CRISPR-edited T cell line that expressed TRBC2^+^ ( Jurkat TRBC2^+^)^15^ (Fig. 2a). In contrast, the JX1.1 antibody failed to stain cell lines with endogenous TRBC1^+^ expression (wild-type Jurkat and H9 cells). Importantly, the JX1.1 antibody staining intensity for TRBC1^+^ cells was similar to that of the CRISPR-edited cell lines with no TCR expression (Jurkat-TCR-knockout (KO) and HPB-ALL TCR-KO)^13,14^ (Fig. 2a). This observation was consistent with the SPR results, which demonstrated a lack of binding of the JX1.1 antibody to the TRBC1 protein (Fig. 1a). We then compared the ability of four anti-TRBC2 antibodies (JX1.1 and the previously described YR3-A5, SAM.2 and KFN) to bind TRBC2^+^ cell lines (wild-type HPB-ALL, MHH-TALL2, HD-MAR-2 and Jurkat TRBC2^+^). Flow cytometry showed that all four antibodies were bound to the cell lines (Fig. 2b,c). The JX1.1 antibody showed the highest median fluorescence intensity when compared to the other anti-TRBC2 antibodies for all four TRBC2^+^ cell lines (Fig. 2c).

Next, we sought to determine whether the JX1.1 antibody could bind to normal T cells that express the TRBC2^+^ TCR. Approximately 60% of normal T cells are TRBC2^+^ cells and ~40% are TRBC1^+^ cells^13–15,17^. A small fraction (1–5%) of T cells express both TRBC1 and TRBC2 due to imperfect allelic exclusion during T cell development and are called dual positive^32,33^ T cells, or do not express either TRBC1 or TRBC2, which are the γδ T cells^34^. T cells from peripheral blood mononuclear cells (PBMCs) were obtained from three different healthy donors. Co-staining with the previously described anti-TRBC1 antibody (clone JOVI.1)^13,15,35^ and the newly described anti-TRBC2 antibody JX1.1 separated the normal T cells into four distinct populations. The anti-TRBC1 antibody classified 29.8–35.7% of normal T cells as TRBC1^+^ cells and the JX1.1 antibody classified 60.9–66.0% of normal T cells as TRBC2^+^ (Fig. 2d,e). Importantly, the vast majority of cells stained with JX1.1 antibody failed to stain with the anti-TRBC1 antibody, demonstrating the lack of binding of the JX1.1 antibody to normal TRBC1^+^ T cells.

**Extended Data Fig. 3 |:**
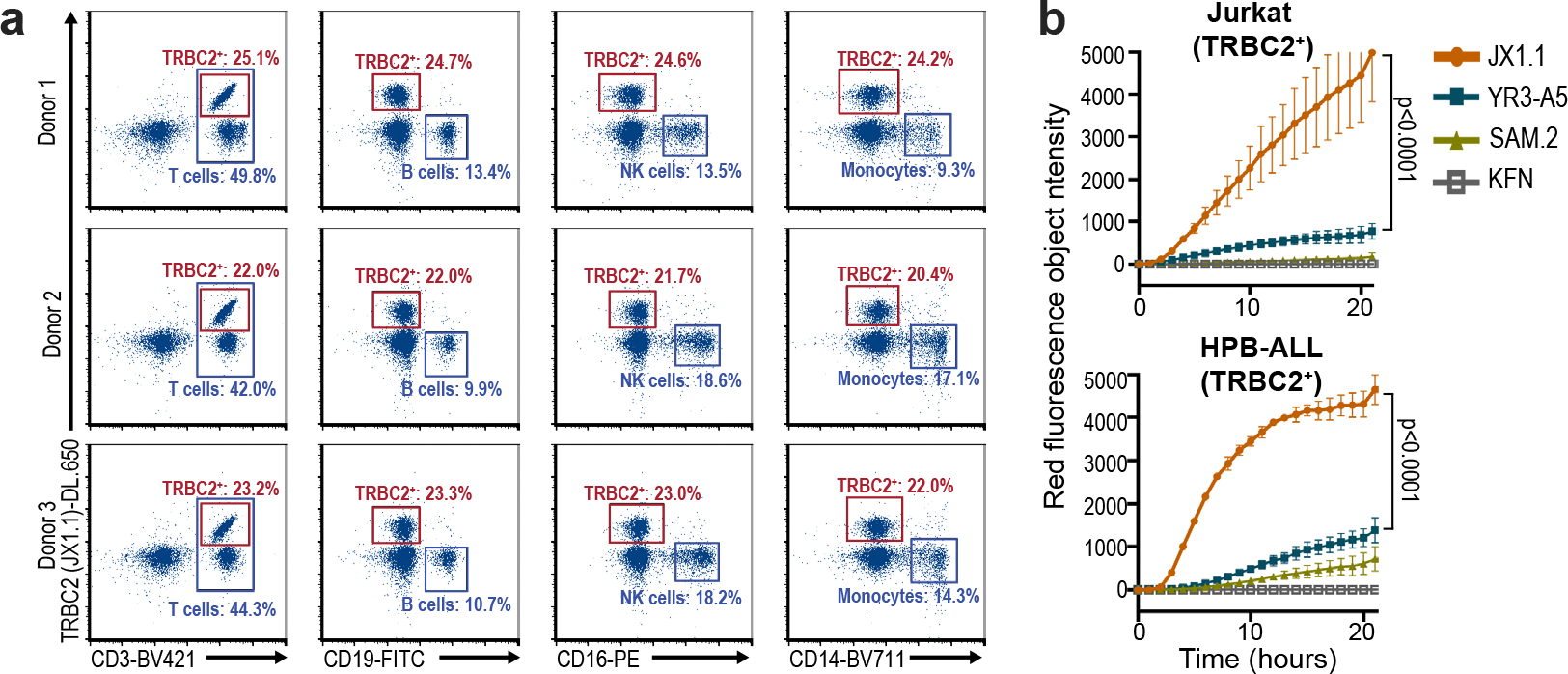
Anti-TRBC2 antibody binding and internalization. **a**, PBMCs isolated from three different normal human donors and stained with the JX1.1 anti-TRBC2 antibody (with C-terminus 6x histidine tag) followed by secondary staining with an anti-histidine DyLight 650 antibody. PBMCs were also stained with the indicated antibodies to identify B cells, NK cells and monocytes. Numbers beside plots indicate the percentage of cells in a representative experiment. **b**, Quantification of red fluorescence over time after the addition of the indicated pHrodo-tagged anti-TRBC2 antibodies. Data represent mean ± standard error of the mean. p-values obtained by one-way ANOVA with Sidak’s multiple comparison test. Data representative of one experiment.

Consistent with expectations, 1.3–2.1% cells were dual positive (TRBC1/2^+^) and bound both antibodies, whereas 1.2–2.9% cells were dual negative (TRBC1/2^−^) and were not stained with either antibody. The YR3-A5 (51.1–62.3%), SAM.2 (35.5–57.9%) and KFN (19.8–37.0%) antibodies identified a lower proportion of cells as TRBC2^+^ (Fig. 2d,e). In addition, staining with YR3-A5, SAM.2 and KFN antibodies did not separate the TRBC1^+^ and TRBC2^+^ cells into distinct populations as well as the JX1.1 antibody (Fig. 2d). To identify possible off-target binding to other immune cells, we incubated the JX1.1 antibody with PBMCs from three different healthy donors. As expected, the JX1.1 antibody stained a subset of CD3^+^ T cells but failed to bind CD19^+^ B cells, CD16^+^ natural killer cells and CD14^+^ monocytes (Extended Data Fig. 3a).

### Anti-TRBC2 antibody is internalized into lysosomes

**Fig. 3 |:**
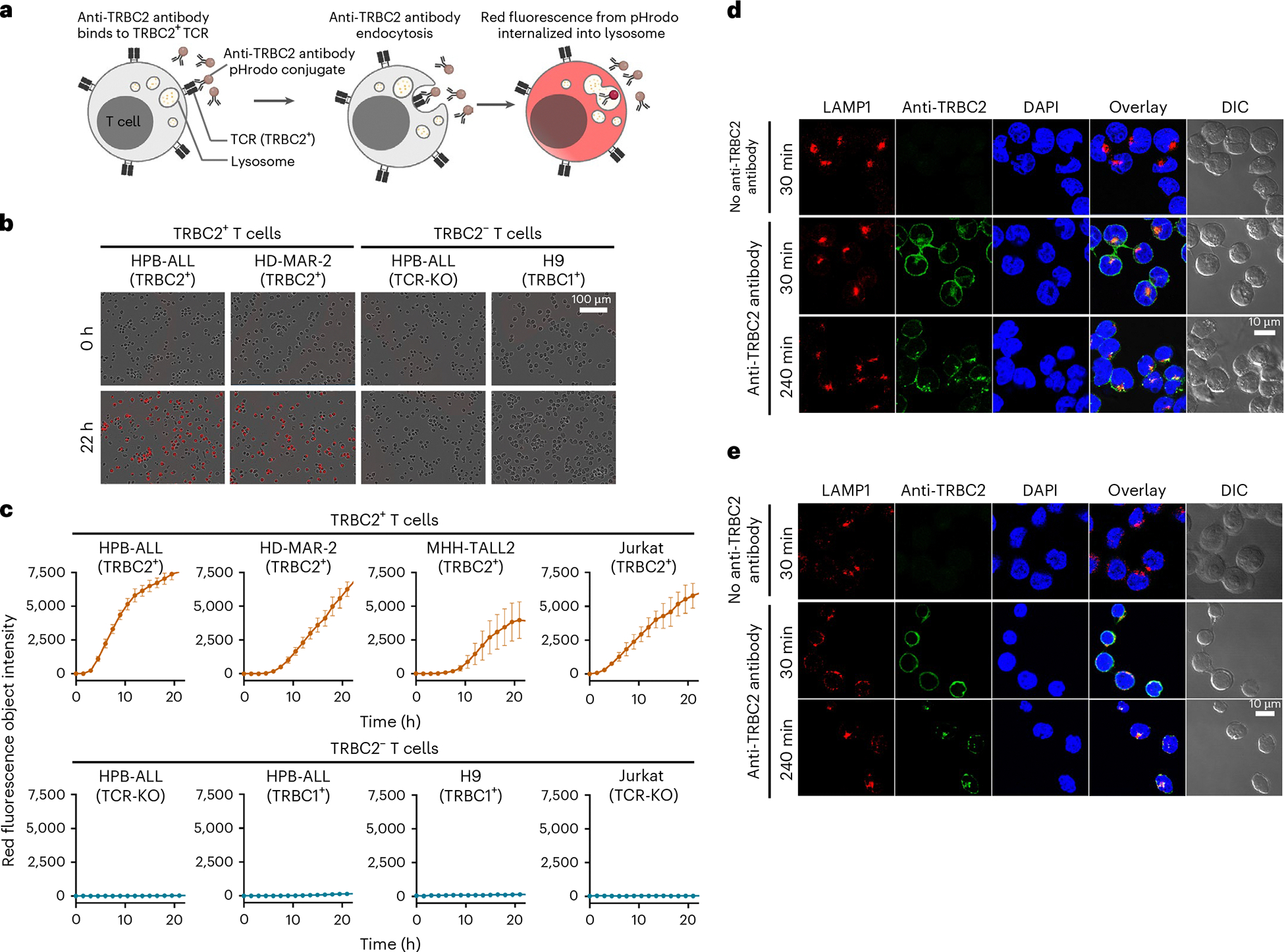
Anti-TRBC2 antibody is internalized into lysosomes. **a**, Schematic showing anti-TRBC2 antibody internalization in TRBC2^+^ T cells. Anti-TRBC2-pHrodo antibody binds to the TRBC2^+^ TCR leading to antibody endocytosis. The anti-TRBC2-pHrodo antibody emits red fluorescence after the fusion of endosome with lysosome. **b**, JX1.1 anti-TRBC2-pHrodo antibody added to TRBC2^+^ cells (HPB-ALL and HD-MAR-2) or TRBC2^−^ cells (HPB-ALL TCR-KO and H9) followed by live cell imaging. **c**, Quantification of red fluorescence over time after the addition of JX1.1 anti-TRBC2-pHrodo antibody to the indicated cell lines. The data represent mean ± s.e.m. from *n* = 3 technical replicates, repeated twice (Jurkat TRBC2^+^ and HPB-ALL-TRBC2^+^) or once (HD-MAR-2, MHH-TALL2, HPB-ALL-TCR-KO, HPB-ALL-TRBC1^+^, H9-TRBC1^+^ and Jurkat-TCR-KO) with similar results. Top: TRBC2^+^ cells. Bottom: TRBC2^−^ cells. **d**,**e**, TRBC2^+^ cells (HPB-ALL-TRBC2^+^ (**d**) and Jurkat TRBC2^+^ (**e**)) were incubated with JX1.1 anti-TRBC2 antibody up to the indicated time points, followed by confocal microscopy. A secondary stain with anti-mouse IgG2a–Alexa Fluor-568 was used to detect the JX1.1 anti-TRBC2 antibody. LAMP1 antibody and DAPI stains mark lysosomes and nuclei, respectively. Scale bars: 10 μm. For **d** and **e**, the number of repeated experiments was two. DIC, differential interference contrast. Icons in **a** created using BioRender.com.

ADCs kill cancer cells through internalization and subsequent release of the cytotoxic payload^6,7^. The TCR is a promising target for ADCs, as TCR activation by peptide–major histocompatibility complex complexes results in rapid internalization of the TCRs^36^. To investigate whether the JX1.1 anti-TRBC2 antibody is internalized, we conjugated the JX1.1 anti-TRBC2 antibody with a pH-sensitive dye (pHrodo). The pHrodo dye emits red fluorescence when exposed to the acidic pH of lysosomes (Fig. 3a). All TRBC2^+^ cells (HPB-ALL, HD-MAR-2, MHH-TALL2 and Jurkat TRBC2^+^) demonstrated a steady increase in red fluorescence after the addition of the JX1.1 anti-TRBC2-pHrodo antibody (Fig. 3b,c). A similar increase in red fluorescence was not observed in TRBC2^−^ cells (HPB-ALL TCR-KO, H9, HPB-ALL TRBC1^+^ and Jurkat-TCR-KO cells) (Fig. 3b,c). The JX1.1 antibody demonstrated a higher rate of internalization when compared to the YR3-A5, KFN and SAM.2 antibodies (Extended Data Fig. 3b). To verify trafficking into lysosomes, TRBC2^+^ cells (HPB-ALL and Jurkat TRBC2^+^) were stained with an antibody against the lysosomal-associated membrane protein 1 (LAMP1). JX1.1 anti-TRBC2 antibody was localized to the cell surface at 30 min and was internalized and co-localized with LAMP1 to the lysosomes at 240 min (Fig. 3d,e). These observations confirmed that JX1.1 is trafficked into lysosomes and can be used to deliver cytotoxic payloads into cells expressing TRBC2.

### Anti-TRBC2 ADC specifically kills TRBC2^+^ cells

**Extended Data Fig. 4 |:**
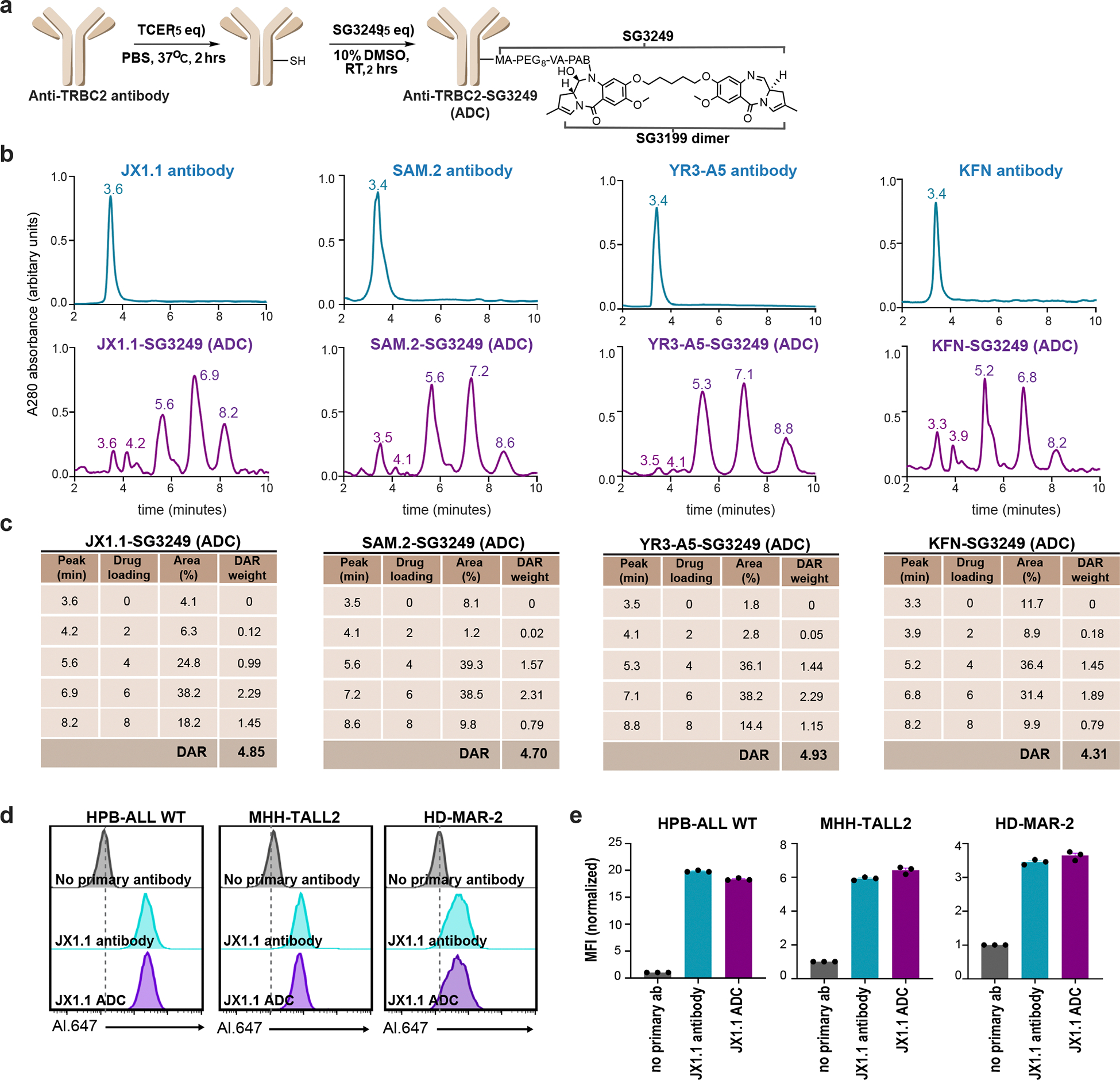
Synthesis and characterization of anti-TRBC2 ADCs. **a**, Schematic of the anti-TRBC2 antibody conjugation to SG3249. **b**, Hydrophobic interaction chromatography of the indicated anti-TRBC2 antibodies and the anti-TRBC2-SG3249 ADCs. **c**, The calculated drug antibody ratio (DAR) for the indicated anti-TRBC1-SG3249 ADCs. Data representative of one (SAM.2, YR3-A5, KFN) or two (JX1.1) independent experiments. **d, e**, Flow cytometry histogram depicting binding of JX1.1 antibody and JX1.1 ADC (1 μg/mL) to the three TRBC2-expressing T-cell lines (**b**) with aggregate median fluorescent intensity from three experiments plotted in (**e**). Data represent mean ± standard error of the mean. The JX1.1 antibody and JX1.1 ADC harbors a C-terminal 6x histidine tag, and were detected using secondary staining with an anti-his Alexa Fluor 647 antibody. Only the anti-his Alexa Flour 647 antibody was used in the no primary antibody condition.

**Fig. 4 |:**
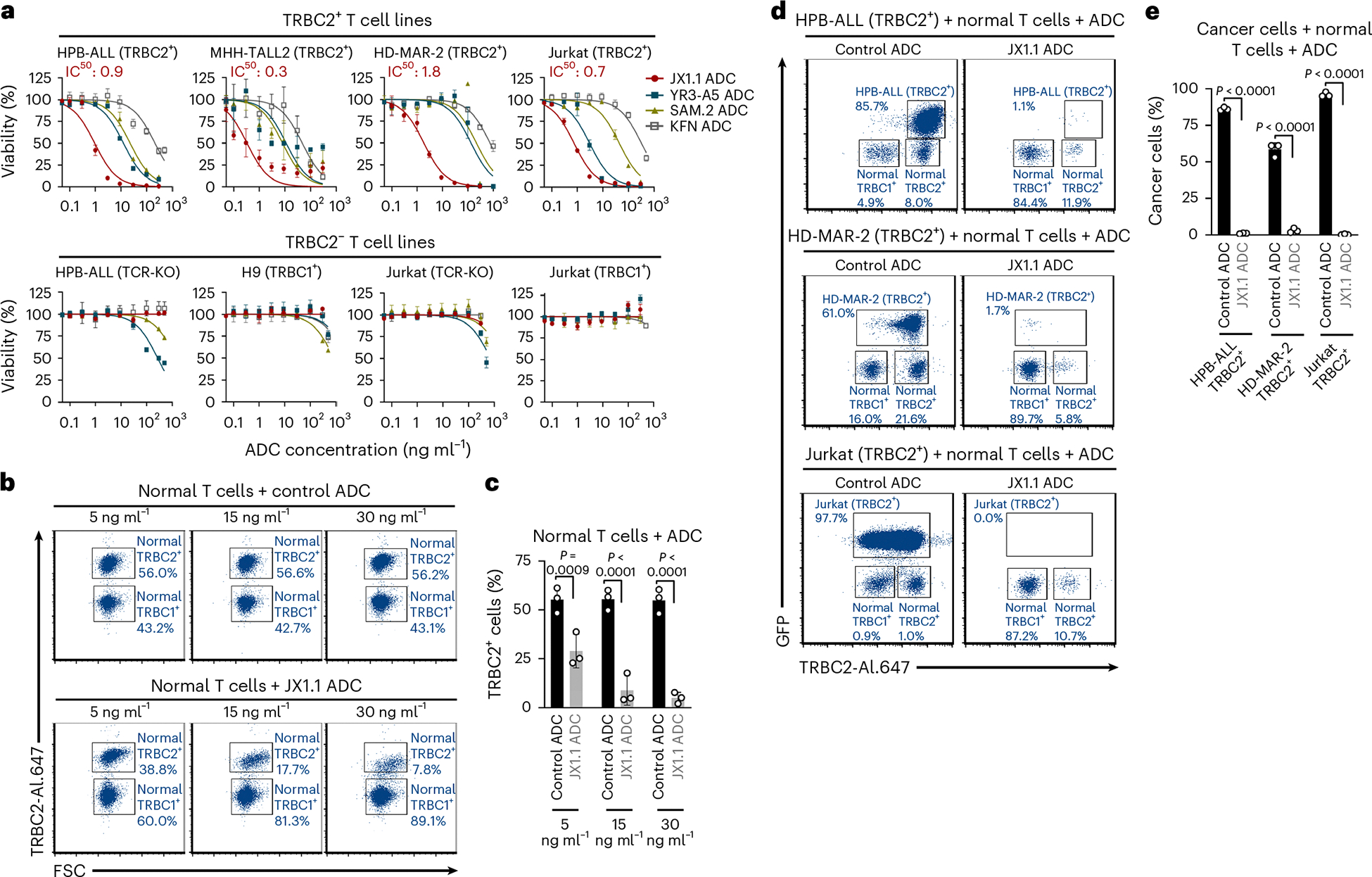
Anti-TRBC2 ADC specifically kills TRBC2^+^ cells. **a**, TRBC2^+^ (HPB-ALL, MHH-TALL2, HD-MAR-2 and Jurkat (TRBC2^+^)) (top) or TRBC2^−^ (HPB-ALL (TCR-KO), H9, Jurkat (TCR-KO) and Jurkat (TRBC1^+^)) (bottom) T cell cancer cell lines incubated with the indicated concentrations of the four anti-TRBC2 ADCs for 5 d. Cancer cell viability was measured by MTS assay (for MHH-TALL2 cells) or by luciferase assay (for all other cells). The IC_50_ for JX1.1 ADC is indicated in the graphs. The data represent the mean ± s.e.m. using *n* = 3 technical replicates from one experiment. **b**,**c**, Normal T cells incubated with IgG2a control ADC (as negative control) (**b**, top) or JX1.1 ADC (**b**, bottom). After 5 d, flow cytometry was used to detect TRBC2^+^ and TRBC1^+^ cells. The numbers beside the plots indicate the percentage of surviving cells (**b**), with data from three human T cell donors shown in **c**. FSC, forward scatter. **d**,**e**, The indicated T cell cancer cell lines cultured with normal T cells in the presence of JX1.1 ADC (**d**) or control ADC (**e**). After 5 d, flow cytometry was used to assess GFP and TRBC2 expression. The numbers beside the plots indicate the percentage of surviving cells in a representative experiment (**d**), with data from three human T cell donors shown in **e**. For **c** and **e**, bar graphs represent the mean ± s.e.m. *P* values were obtained using one-way ANOVA with Šídák’s multiple-comparison test.

SG3249 is a pyrrolobenzodiazepine (PDB) dimer attached to a cleavable linker that crosslinks DNA, leading to cell death, and is used as a payload in ADCs. A CD19-targeting ADC carrying the SG3249 payload received US Food and Drug Administration approval and is in clinical use for the treatment of B cell lymphomas^37^. We conjugated four anti-TRBC2 antibodies to the SG3249 payload to generate ADCs (Extended Data Fig. 4a). After ADC synthesis, hydrophobic interaction chromatography demonstrated a drug-to-antibody ratio (DAR) varying from 4.3 to 4.9 (Extended Data Fig. 4b,c). The JX1.1 antibody and ADC exhibited similar binding patterns to TRBC2^+^ cells (Extended Data Fig. 4d,e), thereby confirming that PDB conjugation did not alter binding to TRBC2 antigen. All four ADCs demonstrated dose-dependent cytotoxicity toward TRBC2^+^ cancer cells (Fig. 4a). The JX1.1 ADC showed substantially higher cytotoxicity (half-maximal inhibitory concentration (IC_50_) 0.3–1.8 ng ml^−1^) than the YR3-A5 ADC (IC_50_ 3.0–133.7 ng ml^−1^), the SAM.2 ADC (IC_50_ 8.0–217 ng ml^−1^) or the KFN ADC (IC_50_ 47.5–750.7 ng ml^−1^) (Fig. 4a and Supplementary Table 2). The observed IC_50_ with the JX1.1 ADC was comparable to the ADCs in clinical use for B and T cell cancers^38,39^. All four anti-TRBC2 ADCs exhibited minimal cytotoxicity toward TRBC2^−^ T cell lines (HPB-ALL (TCR-KO), H9 (TRBC1^+^), Jurkat (TCR-KO) and Jurkat (TRBC1^+^)), demonstrating their specificity (Fig. 4a and Supplementary Table 2).

**Extended Data Fig. 5 |:**
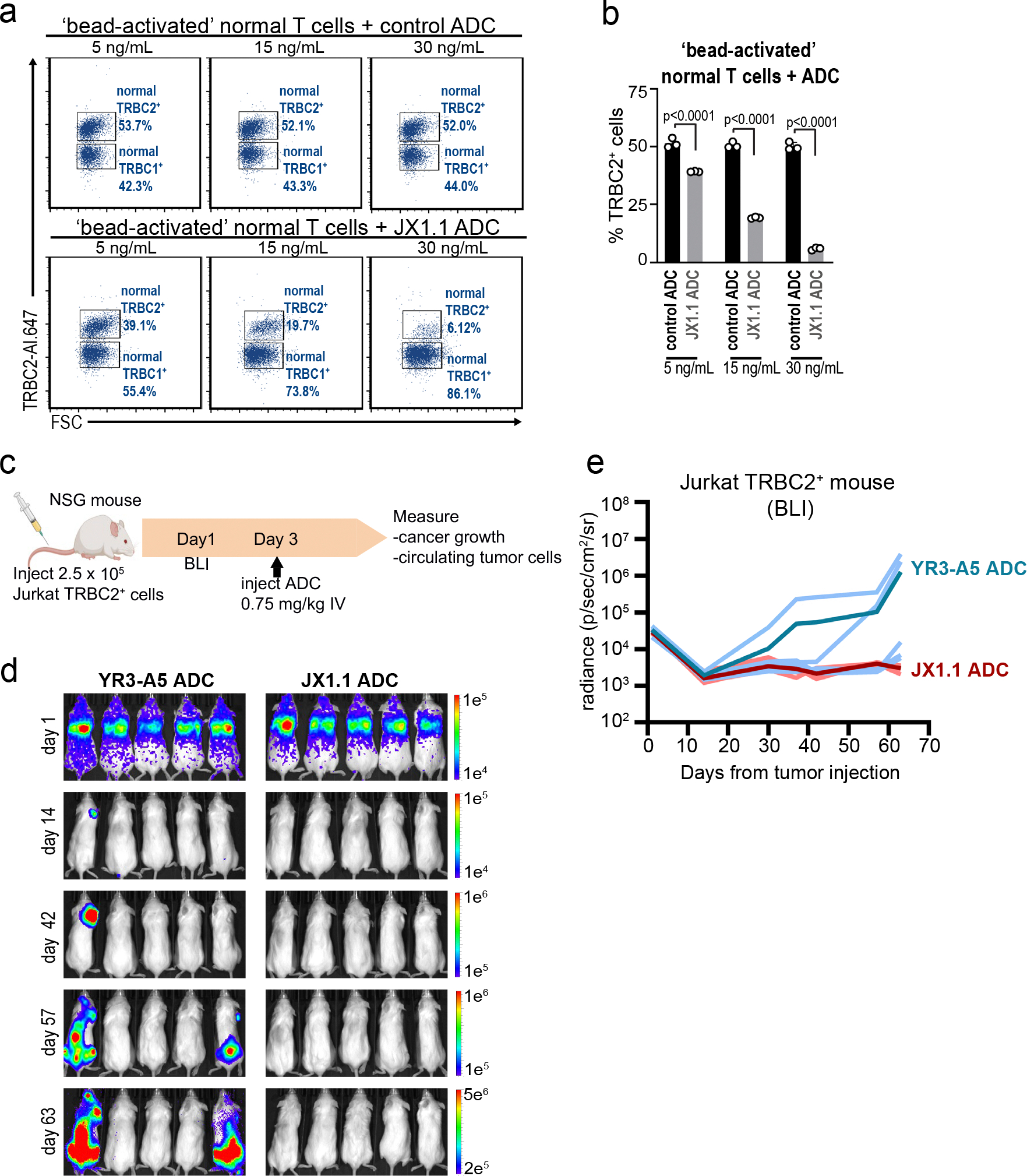
JX1.1 anti-TRBC2 ADC efficacy. **a, b**, normal T cells were activated with CD3/CD28 Dynabeads at a bead:cell ratio of 1:1 for 3 days. Following bead removal, the ‘bead-activated’ normal T cells were incubated with IgG2a control ADC (as negative control) or JX1.1 ADC at the indicated concentrations. After 5 days, flow cytometry was used to detect TRBC2+ and TRBC1+ cells. Numbers beside plots indicate the percentage of surviving cells (**a**), with data from three human T cell donors shown in (**b**). Bar graphs represent mean ± standard error of mean. p-values obtained by one-way ANOVA with Sidak’s multiple comparison test. **c**, Timeline of in vivo experiment using NSG mice injected with Jurkat TRBC2+ cells that express luciferase and GFP. On day 3, mice were intravenously injected with YR3-A5 ADC (5 mice) or JX1.1 ADC (5 mice). **d, e**, BLI was performed on the indicated days with individual mouse data shown in blue (mean value in dark blue) for YR3-A5 ADC, or pink (mean value in red) for JX1.1 ADC (**e**). Panel **c** was created using BioRender.com.

We also observed dose-dependent killing of normal T cells expressing TRBC2, but minimal killing of the normal T cells expressing TRBC1 (Fig. 4b,c and Extended Data Fig. 5a,b). In clinical use, the injected anti-TRBC2 ADC would encounter both normal and malignant T cells in a patient. To mimic this situation, we assessed the JX1.1 ADC cytotoxicity in a mixed culture of normal human T cells and TRBC2^+^ cancer cells (Fig. 4d,e). Specific killing of the TRBC2^+^ T cell cancers (HPB-ALL (TRBC2^+^), HD-MAR-2 (TRBC2^+^) and Jurkat (TRBC2^+^)), along with the TRBC2^+^ normal T cells, with minimal cytotoxicity toward the TRBC1^+^ normal T cells, was observed (Fig. 4d,e).

**Fig. 5 |:**
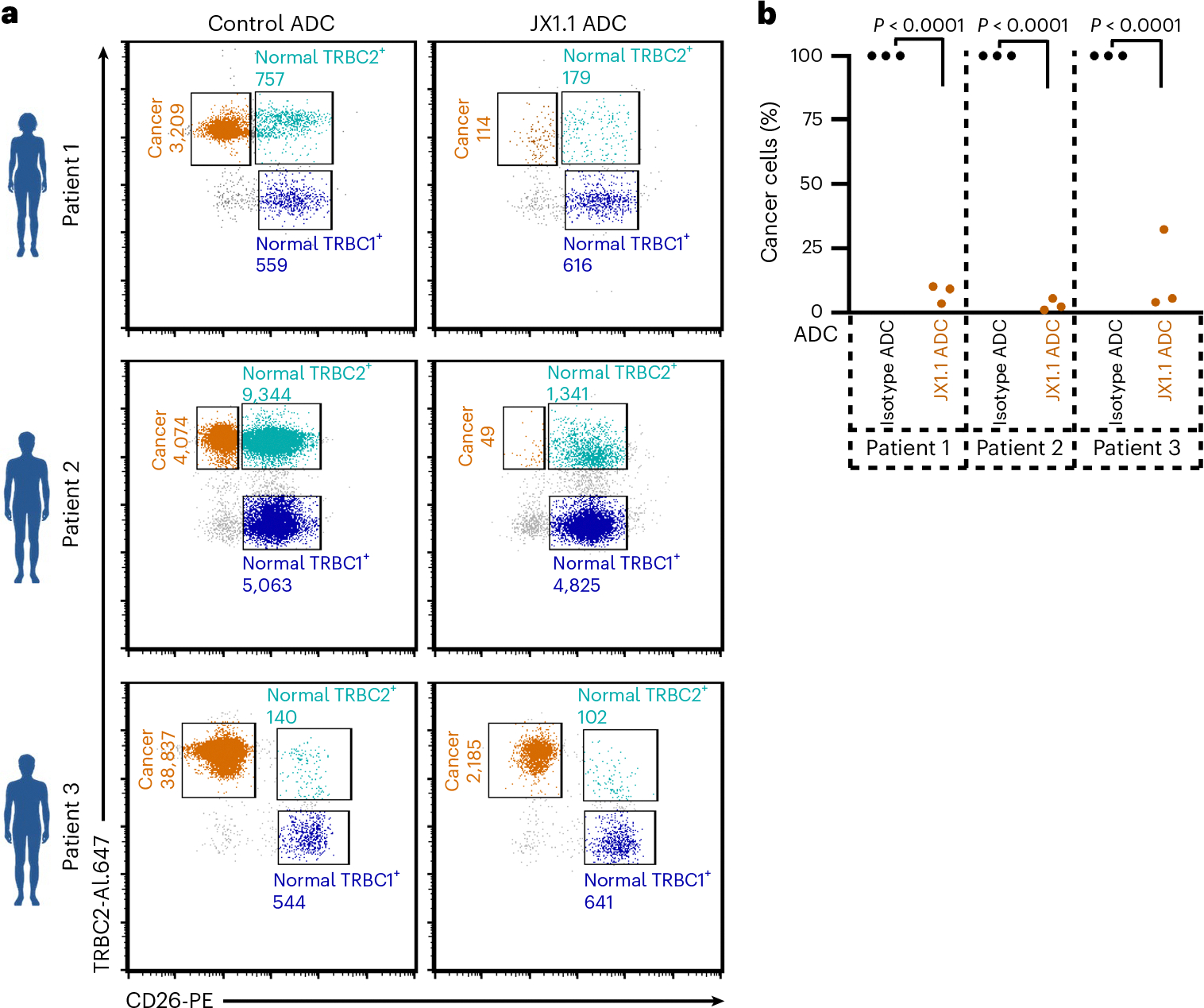
Anti-TRBC2 ADC kills patient-derived cancer cells. PBMCs were isolated from three patients with TRBC2-expressing T cell lymphoma and incubated with either JX1.1 ADC (right) or control ADC (30 ng ml^−1^) (left) for 5 d. **a**, Flow cytometry plots of JX1.1 ADC (right) or control ADC (left) gated on CD3^+^ cells to quantify the number of cancer cells (TRBC2^+^, CD26^−^), normal TRBC2^+^ cells (TRBC2^+^, CD26^+^) and normal TRBC1^+^ cells (TRBC1^+^, CD26^+^). Numbers beside plots indicate cells counted by flow cytometry. **b**, Data from three independent experiments. *P* values were obtained using one-way ANOVA with Šídák’s multiple-comparison test.

We evaluated JX1.1 ADC activity against primary malignant T cells expressing TRBC2 obtained from three patients with CTCLs. We purified PBMCs from the blood of patients with CTCLs. The malignant T cells from these patients lost expression of CD26, which allowed us to differentiate the cancer cells from normal T cells, as previously described^40^. Flow cytometric analysis revealed distinct CD26^−^ and TRBC2^+^ malignant cell populations in all three patients. Patients had varying levels of tumor burden with ~22% (4,074 cancer cells out of a total of 18,481 T cells, in patient 2) to ~98% (38,837 cancer cells out of a total of 39,521 T cells in patient 3) of circulating T cells demonstrating the malignant phenotype (Fig. 5a). Addition of the JX1.1 ADC showed selective cytotoxicity toward the TRBC2^+^ malignant cells and TRBC2^+^ normal T cells with relative sparing of normal TRBC1^+^ cells (Fig. 5a,b).

### Anti-TRBC2 ADC activity in vivo

**Fig. 6 |:**
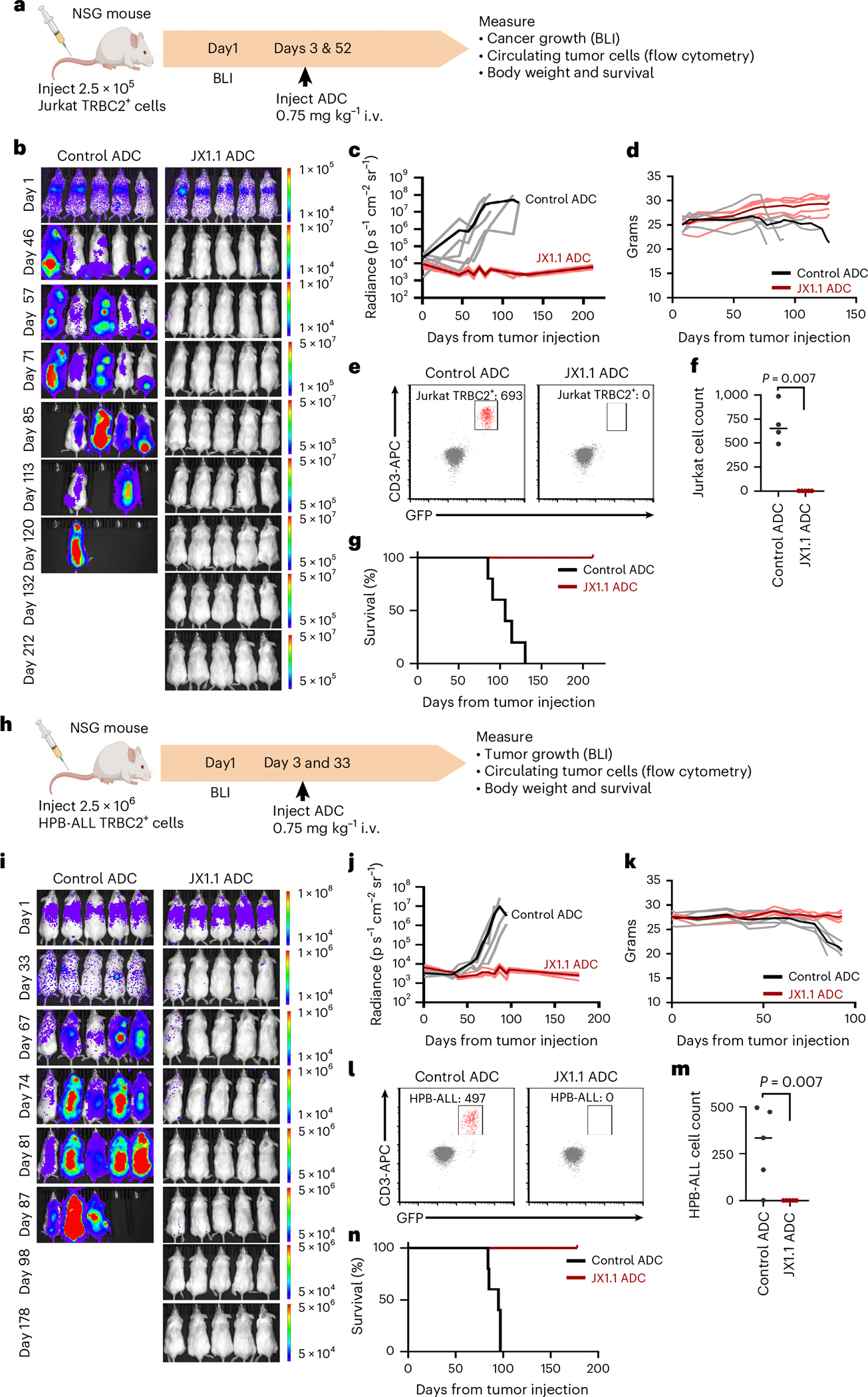
JX1.1 anti-TRBC2 ADC performance in vivo. **a**, Timeline of in vivo experiment using NSG mice injected with Jurkat TRBC2^+^ cells. **b**,**c**, NSG mice intravenously injected with Jurkat TRBC2^+^ cells that express luciferase and GFP, followed by BLI. **b**, On days 3 and 52, mice were intravenously (i.v.) injected with control ADC (5 mice, left) or JX1.1 ADC (5 mice, right). **c**, BLI performed on the indicated days with aggregate data shown. **d**, Body weight of control ADC and JX1.1 ADC-treated mice. **e**, Flow cytometry of mouse blood to assess circulating Jurkat TRBC2^+^ cells (human CD3^+^GFP^+^, shown in red) performed when control ADC-treated mice developed hind-leg paralysis. Left: control ADC; right: JX1.1 ADC. **f**, Aggregate data from flow cytometry for four mice in control ADC and five mice in JX1.1 ADC. **g**, Kaplan–Meier survival curves of NSG mice injected with Jurkat TRBC2^+^ cells, with five mice in each group. Median survival was 106 d in control ADC conditions versus median survival undefined (not reached) in the JX1.1 ADC conditions. *P* = 0.0018 by log(rank) Mantel–Cox test. **h**, Timeline of in vivo experiment using NSG mice injected with HPB-ALL TRBC2^+^ cells. **i**,**j**, NSG mice intravenously injected with HPB-ALL TRBC2^+^ cells that express luciferase and GFP. **i**, On days 3 and 33, mice were intravenously injected with control ADC (5 mice, left) or JX1.1 ADC (5 mice, right). BLI was performed on the indicated days with aggregate data shown in **j**. **k**, Body weight of control ADC and JX1.1 ADC-treated mice. **l**,**m**, Flow cytometry of mouse blood to assess circulating HPB-ALL cells (human CD3^+^GFP^+^, shown in red) performed when control ADC-treated mice developed hind-leg paralysis with aggregate data from five mice in control ADC (**l**, left) and five mice in JX1.1 ADC (**l**, right), also shown in **m**. **n**, Kaplan–Meier survival curves of NSG mice injected with HPB-ALL TRBC2^+^ cells, with five mice in each group. The median survival was 95 d in control ADC conditions versus median survival undefined (not reached) in JX1.1 ADC conditions. *P* = 0.0027 by log(rank) Mantel–Cox test. In **f** and **m**, *P* values were obtained using the two-tailed Mann–Whitney test. In **c**, **d**, **j** and **k**, individual mouse data are shown in gray (mean value in black) for control ADC or pink (mean value in red) for JX1.1 ADC. Icons in **a** and **h** created using BioRender.com.

To assess vivo efficacy, we tested the JX1.1 ADC against TRBC2^+^ T cell cancer xenografts. NOD.Cg- *Prkdc*^*scid*^*Il2rg*^*tm1Wjl*^*/*SzJ (NSG) mice were injected with Jurkat (TRBC2^+^) cancer cells expressing luciferase and GFP (Fig. 6a). Bioluminescence imaging (BLI) confirmed the engraftment of the xenografted cancer cells (Fig. 6b). Mice were then treated with isotype control ADC or the JX1.1 ADC (Fig. 6a). Subsequent BLI demonstrated the expected expansion of cancer cells in mice treated with control ADC (Fig. 6b, left and c). In contrast, no expansion was observed after treatment with the JX1.1 ADC (Fig. 6b, right and c). Cancer expansion led to weight loss in control ADC-treated mice, but weight loss or other signs of toxicity were not observed in JX1.1 ADC-treated mice (Fig. 6d). Cancer regression was confirmed by flow cytometric evaluation of blood cells. Circulating Jurkat cells (human CD3^+^GFP^+^ cells) were detected in the blood of control ADC-treated mice but not in the JX1.1 ADC-treated mice (Fig. 6e,f). The expansion of cancer cells in control ADC-treated mice led to hind-leg paralysis and the mice were euthanized. Hind-leg paralysis was not observed in JX1.1 ADC-treated mice, leading to improved survival (Fig. 6g). We used a second xenograft model with HPB-ALL cells to demonstrate the reproducibility of these in vivo results. HPB-ALL cells expressing luciferase and GFP were xenografted in NSG mice, followed by treatment using control ADC or JX1.1 ADC (Fig. 6h). BLI demonstrated that the JX1.1 ADC substantially reduced tumor burden in mice (Fig. 6i,j). The JX1.1-treated mice maintained stable body weight (Fig. 6k).

**Extended Data Fig. 6 |:**
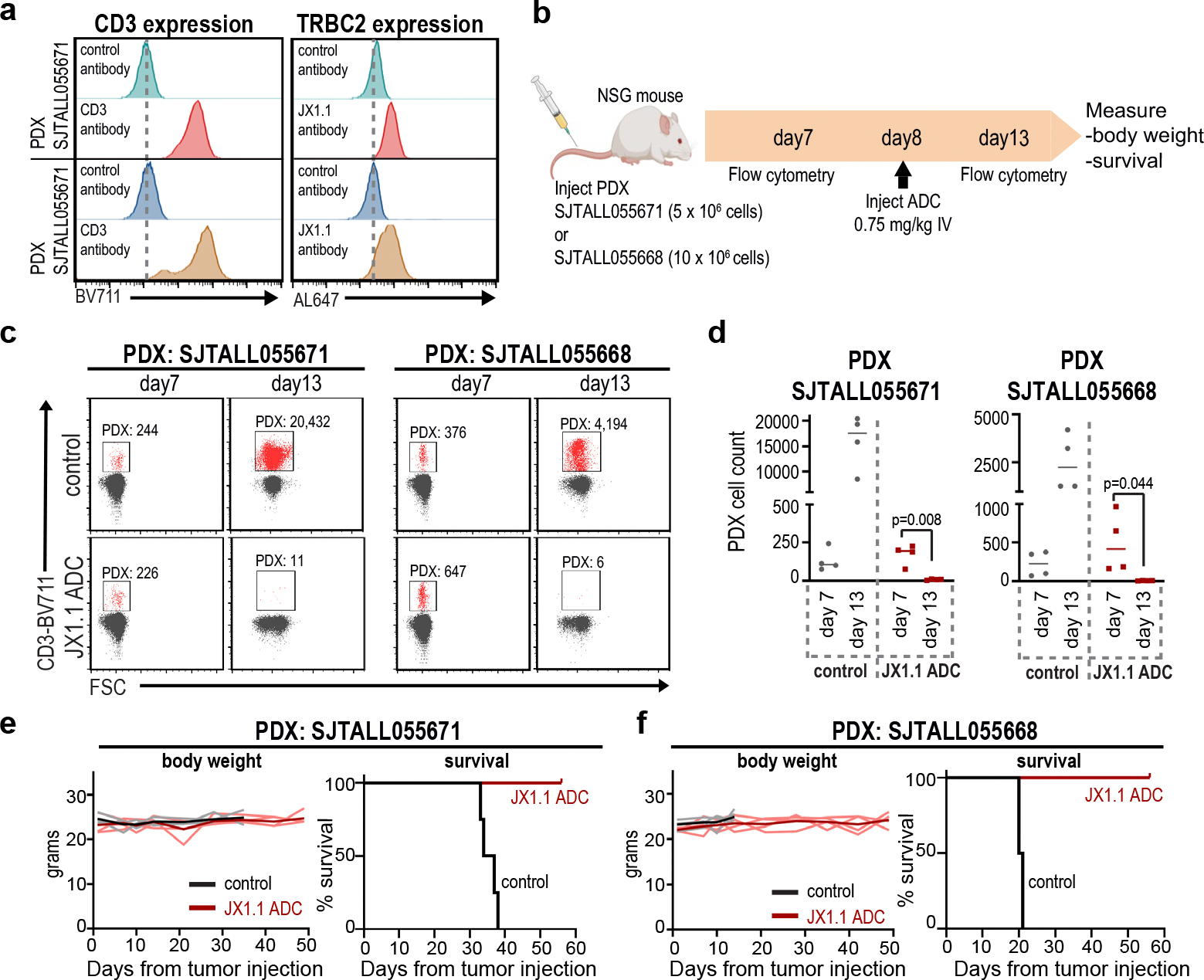
JX1.1 anti-TRBC2 ADC kills patient-derived xenografts. **a**, T cell cancer PDX samples were stained with control antibodies linked with BV711 or AL647 fluorophores or with anti-CD3-BV711 and JX1.1-AL647 antibodies. **b**, Timeline of in vivo experiment using NSG mice injected with PDX cells (8 mice for each PDX). On day 7, mice were intravenously injected with ADCs (control in 4 mice, JX1.1-ADC in 4 mice). **c, d**, Flow cytometry on day 7 and day 13 to assess circulating PDX cells, and aggregate data from 4 mice are shown in (**d**). In (**d**) p-value obtained by one-tailed t-test with Welch’s correction. **e, f**, Body weight and Kaplan-Meier survival of PDX bearing mice with 4 mice in each group. PDX SJTALL056671, median survival 20.5 days in control condition vs. median survival undefined (not reached) in JX1.1 ADC condition. p = 0.0084 by Log-rank Mantel-Cox test. PDX SJTALL055668, median survival 35.5 days in control condition vs. median survival undefined (not reached) in JX1.1 ADC condition. p = 0.0067 by Log-rank Mantel-Cox test. In (**e, f**) individual mouse data are shown in gray (mean value in black) for control, or pink (mean value in red) for JX1.1 ADC. Panel **b** was created using BioRender.com.

Flow cytometry demonstrated circulating HPB-ALL cells (human CD3^+^GFP^+^ cells) in control ADC-treated mice, but HPB-ALL cells were absent in the blood of JX1.1-treated mice (Fig. 6l,m). HPB-ALL cancer expansion led to hind-leg paralysis and death in the control ADC-treated mice, resulting in improved survival in the JX1.1 ADC-treated mice (Fig. 6n). We evaluated the in vivo efficacy of the JX1.1 ADC and the YR3-A5 ADC using NSG mice engrafted with the Jurkat TRBC2^+^ cancer cells. We observed cancer relapses in two out of five mice treated with the YR3-A5 ADC, whereas no relapse occurred in any of the five mice treated with the JX1.1 ADC (Extended Data Fig. 5c–e). Finally, patient-derived xenografts (PDXs) were screened to identify T cell malignancies that express TRBC2. Two PDXs, SJTALL056671 and SJTALL055668, expressed cell-surface CD3, which is part of the TCR complex, and TRBC2 (Extended Data Fig. 6a). To replicate a state of high-disease burden, we injected 5 or 10 million PDX cells into NSG mice (Extended Data Fig. 6b). We confirmed PDX engraftment by flow cytometry on day 7, which was followed by ADC therapy on day 8. After JX1.1 ADC treatment, we observed a decrease in circulating PDX cells as quantified by flow cytometry on day 13 (Extended Data Fig. 6c,d). Weight loss or other signs of toxicity were not observed in JX1.1 ADC-treated mice and mice survived beyond day 50. In contrast, we observed cancer cell expansion in control mice for both PDX models, leading to death (Extended Data Fig. 6c–f).

## Discussion

TRBC1-targeting antibodies and CAR T cells are being developed for the diagnosis^21,41^ and treatment^15,16,42^ of T cell malignancies. We report the generation of a specific and high-affinity anti-TRBC2 antibody that enables the detection and killing of pathogenic TRBC2^+^ cells. The *K*_D_ reflects the rate at which antibody–antigen complexes dissociate over time. The *K*_D_ for the JX1.1 antibody is approximately 2.5-fold lower compared to the YR3-A5 antibody and is one of the contributing factors that render JX1.1 more effective as an ADC. The fraction of remaining antibody–antigen complex after a set duration is represented by *e*^(−*K*D × time)^ as described^43^. Based on this model, approximately 45.7% of JX1.1 is expected to remain bound to the TRBC2 peptide after 10 min (600 s), whereas only about 14.6% of YR3-A5 is expected to remain bound under the same conditions. This increased duration of contact with the TCR may allow increased JX1.1 ADC endocytosis and cytotoxicity^31^ when compared to the other anti-TRBC2 antibodies. Such slow off-rates enabled the successful clinical development of the CD22-targeting immunotoxin for B cell lymphomas^27,28^. The JX1.1 antibody comprises human variable light and heavy chains fused to a murine IgG2a Fc region. For clinical translation, the murine Fc domain will require substitution with a human Fc. JX1.1 exhibits an affinity of approximately 10 nM for the TRBC2 peptide. A CD22-directed antibody fragment with a similar 10-nM affinity and conjugated to an immunotoxin demonstrated complete responses in hairy cell leukemia^28,44^. Thus, the current JX1.1 ADC has the potential to induce cancer regression in human T cell malignancies. Further affinity maturation could potentially enhance therapeutic efficacy; it may also increase cross-reactivity with the TRBC1 peptide. We observed that the SAM.2 clone and clones KFN and KFNM share a similar affinity to the TRBC2 peptide, T cell-staining patterns and antibody internalization kinetics. The antibody sequence of SAM.2 is unknown but it is likely to be similar to KFN or KFNM.

We anticipate that the anti-TRBC2 ADC therapy, like all therapies, may encounter some predictable limitations if applied in patients. ADC-mediated TRBC2 targeting may lead to relapse due to either loss of TCR or TRBC2 expression or acquired resistance to the PBD payload^45^. Patients may experience PBD payload-specific toxicities such as cytopenia, electrolyte imbalances and liver enzyme elevations. Depletion of a substantial fraction of normal T cells may increase the risk of certain infections. However, severe infections were not reported in the phase 1/2 trial of CAR T cells targeting TRBC1 (ref. 16). The TRBV gene selection is independent of TRBC gene selection and, as a result, all TRBV genes are represented in both TRBC1-expressing and TRBC2-expressing cells^21^. Consequently, both TRBC1^+^ and TRBC2^+^ cells harbor TCRs specific for different viral peptides^13^. The normal CD4 count ranges between 500 cells per μl and 1,500 cells per μl and opportunistic infections arise in patients with human immunodeficiency virus (HIV) when the CD4^+^ count is <200 cells per μl (ref. 46). Similarly, normal CD8^+^ cell count ranges between 150 cells per μl and 1,000 cells per μl and reports suggest that CD8^+^ count >10–50 cells per μl is sufficient for antiviral responses^47,48^. TRBC2-targeting therapies deplete ~60% of T cells and thus 200–360 CD4^+^ cells per μl and 60–400 CD8^+^ cells per μl are expected to persist post-therapy. Although CD4^+^ and CD8^+^ counts are expected to remain above the minimum threshold for immune function, some patients may experience lower cell counts and be at risk for infections, requiring antimicrobial prophylaxis.

Targeting the cancer-associated, clonal TRBV segments provides an alternative method of selectively killing the T cell cancers^14,49^. TRBV targeting is advantageous because it preserves a higher fraction of normal T cells and patients may experience lower treatment-related immunosuppression. However, TRBV targeting also requires the generation and testing of a large number of therapeutic antibodies to cover the >30 different TRBV gene segments. Finally, it is worth noting that TRBC targeting may be relevant to serious illnesses in addition to T cell malignancies. Oligoclonal self-reactive T cells are known to drive several human leukocyte antigen-linked pathologies such as celiac disease^50,51^, autoimmune arthritis^52–56^, Behçet’s disease^57,58^, vitiligo^59^ and graft-versus-host disease^60^. TRBC-directed depletion may be beneficial in these disorders if the self-reactive TCRs share identical TRBC segments.

## Methods

The research complies with all relevant ethical regulations, and the study protocol was approved by the Johns Hopkins Institutional Review Board (IRB, protocol no. IRB00247891) and the JHU Animal Care and Use Committee (protocol no. MO21M43). Randomization was not applicable to in vitro experiments and during ADC synthesis or characterization, because it was necessary to know the identity of the samples to interpret the data. For confocal microscopy experiments, images were taken at random locations on slides. For in vivo studies, mice were randomized into different treatment groups after tumor injection on day 0. Data collection and analysis were not performed blind to the conditions of the experiments. No animals were excluded from analysis. One mouse in the control ADC condition died due to cancer progression before blood collection and was thus not included in the flow cytometry plot in Fig. 6f. Further information on research design is available in the Reporting Summary linked to this article.

### Primary human T cells and cell lines

Ficoll Paque Plus (GE Healthcare, cat. no. GE17–1440–02) density gradient centrifugation was used to isolate PBMCs from leukapheresis products (Stemcell Technologies) that were collected in the United States and IRB approved. PBMCs were treated with Human T-Activator CD3/CD28 Dynabeads (Thermo Fisher Scientific, cat. no. 11131D) for 3 d at a bead-to-cell ratio of 1:1 to expand and isolate human T cells. T cells were maintained in Roswell Park Memorial Institute (RPMI) 1640 medium with 10% fetal bovine serum (FBS; GE Healthcare, cat. no. SH30071.03), 1% penicillin–streptomycin (Thermo Fisher Scientific), recombinant human interleukin 2 (IL-2; 100 IU ml^−1^) (aldesleukin, Prometheus Therapeutics and Diagnostics) and recombinant human IL-7 (5 ng ml^−1^) (BioLegend, cat. no. 581906). HPB-ALL (Deutsche Sammlung von Mikroorganismen und Zellkulturen (DSMZ)), MHH-TALL2 (DSMZ), HD-MAR-2 (DSMZ), H9 (American Type Culture Collection (ATCC)) and Jurkat Clone E6–1 (ATCC) were maintained in RPMI-1640 (ATCC, cat. no. 30–2001) supplemented with 1% penicillin–streptomycin and 10% HyClone FBS (GE Healthcare, cat. no. SH30071.03). The cells were cultured in a humidified incubator at 37 °C with 5% CO_2_. Luciferase and GFP were expressed using retroviral transduction in the desired cell lines as described. Cell lines were obtained from ATCC or DSMZ, where they were authenticated with short tandem repeat profiling. In addition, cell-line authenticity was confirmed with immunophenotyping that matched the reports at ATCC and DSMZ. We obtained human T cell cancers from patient samples stored at the Johns Hopkins biorepository under an approved protocol (see above). Patients provided informed consent and did not receive compensation. Sex and gender were not considered relevant for the study design.

### Cell staining, flow cytometry and cell sorting

Cell suspension and staining methods have been described previously^15^. Stained cells were analyzed using an Attune NxT (Thermo Fisher Scientific), an Attune CytPix (Thermo Fisher Scientific) or an IntelliCyt iQue Screener PLUS (Sartorius). BD FACSAria II was used for cell sorting. The following antibodies were used for flow cytometry: allophycocyanin (APC) anti-human CD3 (clone OKT3, BioLegend, cat. no. 317318), phycoerythrin (PE) anti-human CD4 (clone RPA-T4, BioLegend, cat. no. 300508), BV-421 anti-human CD3 (clone UCHT1, BioLegend, cat. no. 300434), BV711 anti-human CD3 (clone OKT3, BioLegend, cat. no. 317328), PE anti-human Cβ1 TCR (clone JOVI.1, BD Biosciences, cat. no. 565776), APC anti-human CD7 (clone CD7–6B7, BioLegend, cat. no. 343108), APC anti-human CD26 (clone BA5b, BioLegend, cat. no. 302710), BV711 anti-CD14 (clone MφP9, BD, cat. no. 563372), PE anti-CD16 (clone B73.1, BioLegend, cat. no. 360704) and FITC anti-CD19 (clone HIB19, Thermo Fisher Scientific, cat. no. 11–0199-42). The following viability dye was used to gate out dead cells: LIVE/DEAD Fixable Near-IR Kit (Invitrogen, cat. no. L10119). The following secondary antibodies were used: Alexa Fluor-647 anti-His Tag antibody (BioLegend, cat. no. 362611), goat anti-mouse IgG2a–Alexa Fluor-488 (Thermo Fisher Scientific, cat. no. A-21131), goat anti-mouse IgG–Alexa Fluor-647 (Thermo Fisher Scientific, cat. no. A55060) and DyLight 650 anti-6X His tag antibody (clone AD1.1.10, abcam, cat. no. ab117504). For primary and secondary antibody stain, 5 μl of antibody was added to 100 μl of total staining solution (1:20 dilution). Flow cytometry data were analyzed with either the FlowJo v10.1 software, iQue Forecyt Software (Sartorius) or the BD FACSDiva Software.

### Phage library preparation

The six CDRs in YR3-A5 scFv were annotated by the abYsis tool using the Kabat region definition^61^. We identified 74 variant sites within the 6 CDR regions and neighboring residues with unusual amino acids (such as low frequency in human CDR sequences) and excluded amino acids with low diversity at that site. The original amino acid was replaced with 1 of the other 19 amino acids for a total of 1,406 variants. The site saturation variant library, consisting of 1,406 variants + 1 (the parent sequence), was synthesized by Twist Biosciences followed by cloning into the pADL-10b phagemid vector (Antibody Design Labs). Next-generation sequencing confirmed the production of the single variant library. The phagemid vector containing the single variant libraries was reconstituted in sterile water, diluted and 60 ng in 30-μl volume of each library was combined with 20 μl of SS320 electrocompetent cells on ice. Half of the mixture (25 μl) was added to a Gene Pulser electroporation cuvette (BioRad), collected to the bottom by gentle tapping and electroporated at 200 Ω, 25 mF and 1.8 kV. Immediately after electroporation, 660 μl of recovery medium (Lucigen) was used to resuspend the bacteria and transferred to a 1.5-ml skirted twist cap tube, and the bacteria were incubated for 45 min at 37 °C with shaking at 250 rpm.

Subsequently, the recovered bacteria were plated on a 24 × 24 cm^2^ 2×YT agar plate supplemented with 100 μg ml^−1^ of carbenicillin and 2% glucose and incubated for 6 h at 37 °C. After incubation, transformed bacteria were scraped with 10 ml of 2×YT medium (Sigma-Aldrich) and diluted to 2.5 × 10^9^ cells ml^−1^ in 2×YT supplemented with 20% glycerol. The resultant mixture was aliquoted into a 1.5-ml skirted twist cap tube (1–1.5 ml each) and stored at −80 °C. Each phage library was grown by diluting enough of its corresponding glycerol stock (based on calculated stock concentration) to obtain approximately 2.5 × 10^9^ SS320 cells in 50 ml of 2×YT with 100 μg ml^−1^ of carbenicillin and 2% glucose (optical density at 600 nm (OD_600_) =0.05) at 37 °C with 250 rpm shaking to reach mid log(growth phase) (OD_600_ of approximately 0.4–0.5), at which point the culture was infected with 50 μl (1 × 10^11^ phage particles) of M13KO7 helper phage (Antibody Design Labs) for an approximate multiplicity of infection of 4. The culture was again incubated at 37 °C for 1 h with shaking at 250 rpm, after which it was centrifuged at 3,000*g* for 10 min. The supernatant was discarded and the culture was resuspended in 100 ml of 2×YT medium with 100 μg ml^−1^ of carbenicillin, 50 μg ml^−1^ of kanamycin and 50 μM isopropyl-β-D-thiogalactopyranoside (Sigma-Aldrich) and incubated overnight at 30 °C shaking at 250 rpm. The bacteria were removed by centrifugation at 3,000*g* for 10 min and the supernatant was transferred to a new tube and centrifuged again at 12,000*g* for 12 min at 4 °C. Phage in the final supernatant was precipitated with the addition of 20% PEG 8000 + 2.5 M NaCl to a final concentration of 4% PEG 8000 + 0.5 M NaCl and incubated on ice for 40 min. The precipitated phage was centrifuged at 12,000*g* for 40 min at 4 °C, the supernatant discarded and phage pellets were resuspended in 1 ml of Tris-buffered saline with 2 mM EDTA containing 0.1% sodium azide. Phage libraries were diluted, mixed 1:1 with mid log(phase) SS320 cells and plated on 2xYT agar plates supplemented with 100 μg ml^−1^ of carbenicillin and 2% glucose for determination of the phage titer.

### Phage selection

To select high-affinity phage clones that bind TRBC2^+^ cells, we modified the SLISY^30^ as follows. Target cells were fixed with 1% paraformaldehyde and blocked with 3% bovine serum albumin (BSA) before and during exposure to phage libraries. Isogenic TRBC1-expressing and TRBC2-expressing HPB-ALL cells were generated as described previously^15^. Each cell line was washed once with phosphate-buffered saline (PBS) and then fixed with 1% paraformaldehyde + PBS solution at a concentration of 1 × 10^6^ cells ml^−1^. The cells were gently mixed and incubated at room temperature for 20 min in the dark. After fixation, the cells were washed twice with 50 ml of PBS, resuspended in 3% BSA at 1 × 10^6^ cells ml^−1^ and incubated at 4 °C for 1 h. The cells were then pelleted and resuspended in 3% BSA at 5 × 10^6^ cells ml^−1^.

Aliquots of 1 ml were transferred into separate wells of a 96-well plate, with each well having a capacity of 2 ml. Phages (approximately 1 × 10^11^ colony-forming units per well), grown as described above, were added to the wells containing cells. The plate was sealed with a sterile AxyMat rubber plate seal and incubated at 4 °C for 1 h with rotation. Phage pools were exposed to the cell lines in separate wells, allowing for downstream comparative analysis of phage binding, which was quantified by sequencing bound phage clones. After incubation, cells were washed 10× with 1 ml of PBS per well, using gentle pipetting (5× per wash). After washing, cells were resuspended in 75 μl of Lucigen QuickExtract and transferred to PCR strips. The cells were heated at 65 °C for 6 min, vortexed for 30 s and then heated again at 98 °C for 2 min. The lysates were applied to Qiashredder spin columns and spun at 18,213*g* in a tabletop centrifuge.

### Phage detection by sequencing

An Illumina MiSeq system was used for multi-read sequencing of the ~700-bp scFv by MPS. As 700 bp exceeds the capacity of MiSeq read lengths, we modified the Illumina standard software and methods so that it could employ the following primers (5′–3′) to sequentially amplify the entire scFv sequence from phage:

clD2_Amp_F2:CGACGTAAAACGACGGCCAGTNNNNNNNNNNNNNNGGGTGAACCTGCAAGCATTA

Phage_Amp_Rev2:CACACAGGAAACAGCTATGACCATGTAACGGTAACCAGGGTGCC

PCR amplification was performed in 25-μl reactions using KAPA HiFi HotStart ReadyMix (Roche) with 5 μM primers and 2.5 μl of template with the following cycles: 98 °C 45 s, then 18 cycles at: 98 °C 15 s, 60 °C 30 s and 72 °C 60 s; then 72 °C 1 min, hold 4 °C. Library preparation was performed as previously described with four cycles of amplification^62^. Samples were evaluated with eight sequencing reads with number of read cycles shown in parentheses using the indicated number of read cycles. *Represents a phosphorothioate linkage:

ACAC read 1 (M13): CGACGTAAAACGACGGCCA*G*T (88)

clD2_L2_F_Primer: GGTCAGTCACCGCAG*C*T (31)

clD2_L3_F2: GAAGCCGAAGATGTTGGTGTGTATT (32)

clD2_H1_F_Primer: GCAAGCGTTAAAGTTAGCTGTAA*A*G (128)

clD2_H2_F2: CTGGTCAAGGTCTGGAATGGAT (55)

clD2_H3_F: CGATAAAAGCACCACCACAGCATAT (99)

M13 index 1: CATGGTCATAGCTGTTTCCTGTG*T*G (10)

Index 2: AATGATACGGCGACCACCGAGATCTACAC (10).

Sequencing results were identified by index and unique identifier as previously. Truncated nucleotide sequences were translated into amino acids using Python (v3.7.17) in JupyterLab notebook (v1.2.6) via Anaconda3 Navigator (v1.9.12). Sequences with one or zero amino acid alterations were used for binding and enrichment analysis. After obtaining the number of reads for each clone, we generated a 95% confidence interval based on an approximated normal distribution (the upper and lower bounds were calculated by the number of reads ±2× square root of reads, respectively). To increase stringency, we used the upper bound for the reads from the TRBC1^+^ cells and the lower bound for the reads from the TRBC2^+^ cells. The phage clones were ranked based on the binding ratio and enrichment ratio as described in Fig. 1b and Extended Data Fig. 2a.

### Antibody production

The JX1.1, KFN and YR3-A5 antibodies were generated as previously described^15^. Briefly, the antibody heavy and light chains were cloned into the pcDNA3.4 expression vector. Antibodies were expressed using ExpiCHO cells, or CHO-S cells (by Thermo Fisher Scientific or GeneScript) and purified using HisTrap column (Millipore Sigma) or AmMag Protein A Magnetic Beads (GeneScript, cat. no. L00695). Preparative SEC was performed with HiLoad 26/600 Superdex 200 pg of 320 ml (Cytiva, cat. no. 28989336) at a flow rate 3.2 ml min^−1^. For analytical SEC, samples were filtered using a 0.2-μm membrane, followed by injection of ~20 μg of antibody in a TSKgel G3000SWxl column, 7.8 mm × 300 mm, particle size 5 μm (TOSOH, cat. no. 0008541) and TSKgel Guard Column SWxl, 6.0 mm × 40 mm (TOSOH, cat. no. 0008543), using 0.1 mol l^−1^ of Na_2_SO_4_ in 0.1 mol l^−1^ of phosphate buffer, pH 6.7 ± 0.3 as the mobile phase, at 0.7 ml min^−1^ flow rate. High-performance liquid chromatography (HPLC) was performed using an Agilent 1260 HPLC or Waters ACQUITY Arc Bio UHPLC. Sodium dodecylsulfate–polyacrylamide gel electrophoresis was performed using 4–20% gradient gel (ExpressPlus GeneScript, cat. no. M42012C) and proteins were stained by Coomassie (GeneScript, cat. no. L00657, eStain). The antibodies were stored in PBS, pH 7.2 at −80 °C. The SAM.2 antibody was purchased from Thermo Fisher Scientific (cat. no. 704905).

### SPR

**Extended Data Fig. 7 |:**
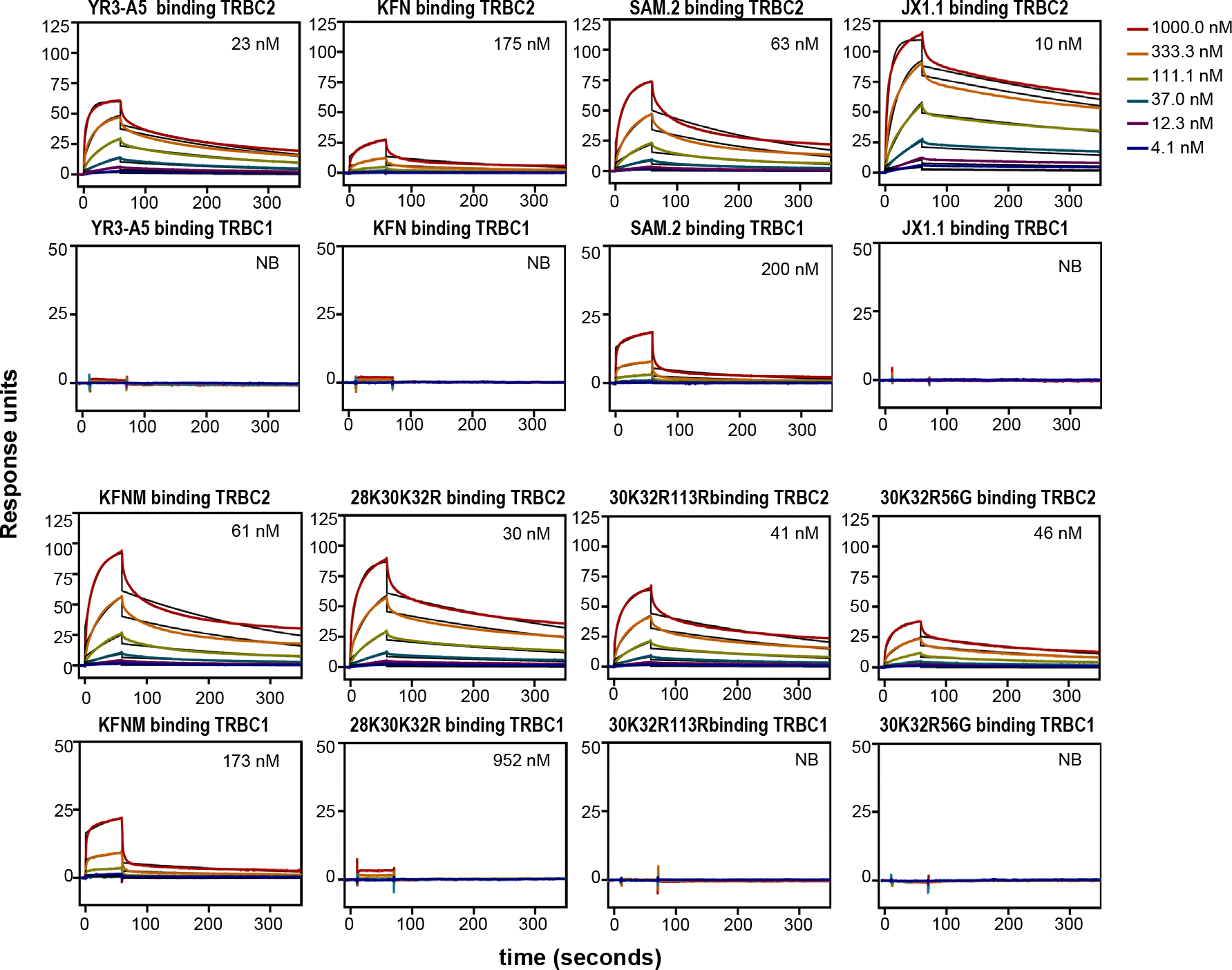
SPR analysis of anti-TRBC2 antibodies. SPR analysis of anti-TRBC2 antibodies binding to the indicated TRBC peptides, with overlaid fitted data shown in black. Data representative of one (KFNM, 28K30K32R, 30K32R113R, 30K32R56G) or two (YR3-A5) or three (KFN, SAM.2, JX1.1) independent experiments.

The sensorgrams were measured with a Biacore T200 (Cytiva) instrument at 25 °C. Protein A/G (50 μM stock concentration) was diluted (1:50 dilution, 1 μM diluted concentration) in 10 mM sodium acetate buffer, pH 4.5 and immobilized on to all flow cells (FCs) to a level of ~2,800 relative units, using the standard amine coupling. The anti-TRBC2 antibodies (stock concentration 0.5 mg ml^−1^) were diluted (1:100 dilution, 0.005 mg ml^−1^ of diluted concentration) in PBS-P and captured on CM5 chips. TRBC1 (Acro Biosystems, cat. no. TR1-H52H4) and TRBC2 (Acro Biosystems, cat. no. TR2-H52H3) proteins were used as analytes to flow over the ligand-captured surfaces. Overnight kinetics were performed for all analytes in the presence of 20 mM phosphate buffer, pH 7.4, 137 mM NaCl, 2.7 mM KCl and 0.05% v:v surfactant P20 (PBS-P). The flow rate of analyte solutions was maintained at 50 μl min^−1^. The contact and dissociation times used were 60 s and 300 s, respectively. Two 20-s pulses of 1:500 H_3_PO_4_ + ddH_2_O (v:v) (85% H_3_PO_4_, Mallinckrodt, cat. no. 2796) were injected for surface regeneration. Multiple-cycle kinetics were performed by injecting threefold dilutions of the analytes from 1,000 nM to 4.1 nM. Sensorgrams were fitted to the 1:1 kinetics binding model (Extended Data Fig. 7) using Biacore T200 evaluation software v3.2.1.

### Confocal microscopy

The pHrodo iFL Red Microscale Protein Labeling Kit (Thermo Fisher Scientific, cat. no. P36014) was used to label the anti-TRBC2 antibody as previously described^15^. Cells were plated on 96-well plates, followed by the addition of anti-TRBC2-pHrodo antibody (10 μg ml^−1^ final concentration). Incucyte SX5 (Sartorius) was used to acquire live cell imaging. Integrated red fluorescence was quantified by summing all red fluorescence intensity within the cell multiplied by the pixel area. For co-localization of anti-TRBC2 antibody and lysosomes, cells were incubated with anti-TRBC2 antibody (1:100 dilution) for the indicated times, followed by a secondary stain with anti-mouse IgG2a–Alexa 568 antibody (1:400, Thermo Fisher Scientific, cat. no. A-21134). The anti-LAMP1–Alexa 647 antibody (1:200) was used to stain lysosomes. Cell staining, slide preparation and image acquisition using a Zeiss LSM700 confocal microscope have been previously described^15,63–66^. Images were adjusted for contrast and cropped in the Zen software (Zeiss).

### ADC generation

ADC production was performed as described previously^15^. Briefly, fivefold molar excess tris(2-carboxyethyl)phosphine hydrochloride (Thermo Fisher Scientific, cat. no. 20490) at 37 °C for 2 h with rotation was used to partially reduce the antibodies. Tris(2-carboxyethyl) phosphine hydrochloride was removed using Zeba Spin 7K molecular weight cut-off (MWCO) columns. The SG3249 (MedChemExpress, cat. no. HY-128952) drug-linker solution was prepared in 10 mM dimethyl sulfoxide stock solution and added at fivefold molar excess to the conjugation reaction mixture to make a final 10% organic and 90% aqueous solution; this was incubated for 2 h with rotation at room temperature. The excess drug was removed using Zeba Spin 7K MWCO columns. Successful conjugation and removal of the free drug were confirmed using HPLC. HPLC was performed with an Agilent 1260 Infinity I LC system as previously described^15^. The DAR was quantified using the weighted average of the integrated peak densities, as outlined in Extended Data Fig. 4. Baseline correction and analysis of HPLC spectra were performed using OriginPro v10.1.5.132.

### ADC cytotoxicity and co-cultures

For cytotoxicity assays, 1 × 10^4^ target cancer cells in 200 μl of RPMI-1640 medium supplemented with 10% FBS and 1% penicillin–streptomycin were added to 96-well, flat-bottomed, tissue-culture-treated plates. The ADC concentrations are listed in the figure legends. The cells were maintained at 37 °C for 5 d. Cell viability for luciferase-expressing cells was assessed using the ONE-Glo luciferase assay (Promega, cat. no. E6110). Cell viability for cells not expressing luciferase was assessed using the CellTiter 96 AQueous One Solution Cell Proliferation Assay (MTS; Promega, cat. no. G3580). For co-culture assays, 1 × 10^4^ cancer cells were added to 2 × 10^4^ normal T cells in 96-well, flat-bottomed, tissue-culture-treated plates in a 100-μl volume of RPMI medium. The ADC concentrations are listed in the figure legends. The co-cultures were maintained at 37 °C for 5 d. The numbers of normal human T cells and cancer cell lines were quantified by flow cytometry-based GFP expression (for GFP-expressing tumor cell lines) or TRBC2 expression.

### Animal experiments

Female and male NOD.Cg-*Prkdc*^*scid*^ *Il2rg*^*tm1Wjl*^/SzJ (NSG) mice aged 8–10 weeks were acquired from the Animal Resources facility of the Johns Hopkins Sidney Kimmel Comprehensive Cancer Center. Mice were housed with a 12-h light-to-dark cycle at approximately 21–24 °C and 50% humidity and were maintained according to the JHU Animal Care and Use Committee (approved research protocol no. MO21M43). Mice were inoculated with cancer cell lines via tail vein injections. The selected ADCs on the dates indicated in the figure legends were injected via the tail vein. Cancer cells were tracked using bioluminescence and measured using the IVIS system (PerkinElmer) as previously described^14,15^. Mouse cheek bleeds were performed on the days indicated in the figure legends. Mouse blood, 100–200 μl, was collected in microvettes (Sarstedt Inc., cat. no. NC9299309) and blood was processed as described in ref. 14. PDX samples were acquired from the PROPEL repository at the St. Jude Children’s Research Hospital. No statistical methods were used to predetermine sample sizes but our sample sizes are similar to those reported in previous publications^14,15^. Mice were euthanized if they lost >20% of body weight, developed hind-leg paralysis or demonstrated other signs of discomfort such as hunched posture, lack of grooming, labored breathing or lethargy.

### Plotting and statistical analyses

Data plotting and statistical tests were performed using GraphPad Prism v10.2.1. The statistical tests conducted for the individual experiments are indicated in the figure legends. Data distribution was assumed to be normal, but this was not formally tested.

## Extended Data

## Supplementary Material

1

**Supplementary information** The online version contains supplementary material available at https://doi.org/10.1038/s43018-025-01069-z.

## Data Availability

The new anti-TRBC2 antibody sequences described in this work are provided in Supplementary Table 3. The remaining data are available within the article, Supplementary Information and from the corresponding author on request. Source data are provided with this paper.

## References

[1] Sung H Global Cancer Statistics 2020: GLOBOCAN estimates of incidence and mortality Worldwide for 36 cancers in 185 countries. CA Cancer J. Clin. 71, 209–249 (2021). 33538338 10.3322/caac.21660

[2] Siegel RL, Giaquinto AN & Jemal A Cancer statistics, 2024. CA Cancer J. Clin. 74, 12–49 (2024). 38230766 10.3322/caac.21820

[3] Fielding AK Outcome of 609 adults after relapse of acute lymphoblastic leukemia (ALL): an MRC UKALL12/ECOG 2993 study. Blood 109, 944–950 (2007). 17032921 10.1182/blood-2006-05-018192

[4] Bellei M The outcome of peripheral T-cell lymphoma patients failing first-line therapy: a report from the prospective, International T-Cell Project. Haematologica 103, 1191–1197 (2018). 29599200 10.3324/haematol.2017.186577 PMC6029527

[5] Cappell KM & Kochenderfer JN Long-term outcomes following CAR T cell therapy: what we know so far. Nat. Rev. Clin. Oncol. 20, 359–371 (2023). 37055515 10.1038/s41571-023-00754-1 PMC10100620

[6] Paul S Cancer therapy with antibodies. Nat. Rev. Cancer 24, 399–426 (2024). 38740967 10.1038/s41568-024-00690-x PMC11180426

[7] Paul S & Zhou S Six events that shaped antibody approvals in oncology. Front. Immunol. 16, 1533796 (2025). 39995677 10.3389/fimmu.2025.1533796 PMC11847691

[8] Chiesa R Base-edited CAR7 T cells for relapsed T-cell acute lymphoblastic leukemia. N. Engl. J. Med. 389, 899–910 (2023). 37314354 10.1056/NEJMoa2300709

[9] Brudno JN Transient responses and significant toxicities of anti-CD30 CAR T cells for CD30+ lymphomas: results of a phase 1 trial. Blood Adv. 8, 802–814 (2024). 37939262 10.1182/bloodadvances.2023011470 PMC10874855

[10] Kirsch IR TCR sequencing facilitates diagnosis and identifies mature T cells as the cell of origin in CTCL. Sci. Transl. Med. 7, 308ra158 (2015). 10.1126/scitranslmed.aaa9122 PMC4765389 26446955

[11] Asnafi V Age-related phenotypic and oncogenic differences in T-cell acute lymphoblastic leukemias may reflect thymic atrophy. Blood 104, 4173–4180 (2004). 15054041 10.1182/blood-2003-11-3944

[12] Sims JE, Tunnacliffe A, Smith WJ & Rabbitts TH Complexity of human T-cell antigen receptor beta-chain constant- and variable-region genes. Nature 312, 541–545 (1984). 6334238 10.1038/312541a0

[13] Maciocia PM Targeting the T cell receptor beta-chain constant region for immunotherapy of T cell malignancies. Nat. Med. 23, 1416–1423 (2017). 29131157 10.1038/nm.4444

[14] Paul S TCR beta chain-directed bispecific antibodies for the treatment of T cell cancers. Sci. Transl. Med. 10.1126/scitranslmed.abd3595 (2021). PMC8236299 33649188

[15] Nichakawade TD TRBC1-targeting antibody-drug conjugates for the treatment of T cell cancers. Nature 628, 416–423 (2024). 38538786 10.1038/s41586-024-07233-2 PMC11250631

[16] Cwynarski K TRBC1-CAR T cell therapy in peripheral T cell lymphoma: a phase 1/2 trial. Nat. Med. 10.1038/s41591-024-03326-7 (2024). PMC11750712 39528665

[17] Ferrari M Structure-guided engineering of immunotherapies targeting TRBC1 and TRBC2 in T cell malignancies. Nat. Commun. 15, 1583 (2024). 38383515 10.1038/s41467-024-45854-3 PMC10881500

[18] TRBC2 Chimeric Recombinant Mouse Monoclonal Antibody (SAM.2). Thermo Fisher https://www.thermofisher.com/antibody/product/TRBC2-Chimeric-Antibody-clone-SAM-2-Recombinant-Monoclonal/704905 (2024).

[19] McCutcheon M Beckman Coulter Life Sciences achieves industry-first with commercial release of anti-TRBC2 conjugated antibody for flow cytometry. Beckman Coulter https://www.beckman.com/news/commercial-release-of-anti-trbc2-conjugated-antibody-for-flow-cytometry (2024).

[20] Loew A, Katragadda M, Guntas G, Marek P & Palakurthi S Methods of detecting TRBC1 or TRBC2. WIPO (PCT) WO2022047046A1 patent (2022).

[21] Horna P Dual T-cell constant beta chain (TRBC)1 and TRBC2 staining for the identification of T-cell neoplasms by flow cytometry. Blood Cancer J. 14, 34 (2024). 38424120 10.1038/s41408-024-01002-0 PMC10904869

[22] Reff ME Depletion of B cells in vivo by a chimeric mouse human monoclonal antibody to CD20. Blood 83, 435–445 (1994). 7506951

[23] Law CL Expression and characterization of recombinant soluble human CD3 molecules: presentation of antigenic epitopes defined on the native TCR-CD3 complex. Int. Immunol. 14, 389–400 (2002). 11934875 10.1093/intimm/14.4.389

[24] Pescovitz MD Rituximab, an anti-cd20 monoclonal antibody: history and mechanism of action. Am. J. Transplant. 6, 859–866 (2006). 16611321 10.1111/j.1600-6143.2006.01288.x

[25] Uchiyama S Development of novel humanized anti-CD20 antibodies based on affinity constant and epitope. Cancer Sci. 101, 201–209 (2010). 19930155 10.1111/j.1349-7006.2009.01392.x PMC11158754

[26] Wang Z CCR4-IL2 bispecific immunotoxin is more effective than brentuximab for targeted therapy of cutaneous T-cell lymphoma in a mouse CTCL model. FEBS Open Bio. 13, 1309–1319 (2023). 10.1002/2211-5463.13625 PMC10315799 37157185

[27] Alderson RF CAT-8015: a second-generation pseudomonas exotoxin A-based immunotherapy targeting CD22-expressing hematologic malignancies. Clin. Cancer Res. 15, 832–839 (2009). 19188153 10.1158/1078-0432.CCR-08-1456 PMC2742326

[28] Kreitman RJ & Pastan I Antibody fusion proteins: anti-CD22 recombinant immunotoxin moxetumomab pasudotox. Clin. Cancer Res. 17, 6398–6405 (2011). 22003067 10.1158/1078-0432.CCR-11-0487 PMC3201735

[29] Tai YT Novel anti-B-cell maturation antigen antibody-drug conjugate (GSK2857916) selectively induces killing of multiple myeloma. Blood 123, 3128–3138 (2014). 24569262 10.1182/blood-2013-10-535088 PMC4023420

[30] Lu S The rapid and highly parallel identification of antibodies with defined biological activities by SLISY. Nat. Commun. 14, 17 (2023). 36596784 10.1038/s41467-022-35668-6 PMC9808734

[31] Sadekar S, Figueroa I & Tabrizi M Antibody drug conjugates: application of quantitative pharmacology in modality design and target selection. AAPS J. 17, 828–836 (2015). 25933599 10.1208/s12248-015-9766-0 PMC4476995

[32] Balomenos D Incomplete T cell receptor V beta allelic exclusion and dual V beta-expressing cells. J. Immunol. 155, 3308–3312 (1995). 7561023

[33] Davodeau F Dual T cell receptor beta chain expression on human T lymphocytes. J. Exp. Med. 181, 1391–1398 (1995). 7699325 10.1084/jem.181.4.1391 PMC2191978

[34] Groh V Human lymphocytes bearing T cell receptor gamma/delta are phenotypically diverse and evenly distributed throughout the lymphoid system. J. Exp. Med. 169, 1277–1294 (1989). 2564416 10.1084/jem.169.4.1277 PMC2189233

[35] Viney JL, Prosser HM, Hewitt CR, Lamb JR & Owen MJ Generation of monoclonal antibodies against a human T cell receptor beta chain expressed in transgenic mice. Hybridoma 11, 701–713 (1992). 1284120 10.1089/hyb.1992.11.701

[36] Krangel MS Endocytosis and recycling of the T3-T cell receptor complex. The role of T3 phosphorylation. J. Exp. Med. 165, 1141–1159 (1987). 3104527 10.1084/jem.165.4.1141 PMC2188576

[37] Caimi PF Loncastuximab tesirine in relapsed or refractory diffuse large B-cell lymphoma (LOTIS-2): a multicentre, open-label, single-arm, phase 2 trial. Lancet Oncol. 22, 790–800 (2021). 33989558 10.1016/S1470-2045(21)00139-X

[38] Francisco JA cAC10-vcMMAE, an anti-CD30-monomethyl auristatin E conjugate with potent and selective antitumor activity. Blood 102, 1458–1465 (2003). 12714494 10.1182/blood-2003-01-0039

[39] Zammarchi F ADCT-402, a PBD dimer-containing antibody drug conjugate targeting CD19-expressing malignancies. Blood 131, 1094–1105 (2018). 29298756 10.1182/blood-2017-10-813493

[40] Hristov AC, Vonderheid EC & Borowitz MJ Simplified flow cytometric assessment in mycosis fungoides and Sezary syndrome. Am. J. Clin. Pathol. 136, 944–953 (2011). 22095381 10.1309/AJCP09OTJOYAVZZK

[41] Pierog O The real-world application of T-cell receptor constant beta-1 chain antibody assay in cutaneous T-cell lymphoma. Br. J. Haematol. 10.1111/bjh.20060 (2025). 40127907

[42] Shafagati N, Paul S, Rozati S & Sterling CH Antibody-based therapies for peripheral T-cell lymphoma. Cancers 10.3390/cancers16203489 (2024). PMC11506347 39456582

[43] O’Shannessy DJ, Brigham-Burke M, Soneson KK, Hensley P & Brooks I Determination of rate and equilibrium binding constants for macromolecular interactions using surface plasmon resonance: use of nonlinear least squares analysis methods. Anal. Biochem. 212, 457–468 (1993). 8214588 10.1006/abio.1993.1355

[44] Kreitman RJ Efficacy of the anti-CD22 recombinant immunotoxin BL22 in chemotherapy-resistant hairy-cell leukemia. N. Engl. J. Med. 345, 241–247 (2001). 11474661 10.1056/NEJM200107263450402

[45] Corbett S The role of specific ATP-binding cassettetransporters in the acquired resistance to pyrrolobenzodiazepine dimer-containing antibody-drug conjugates. Mol. Cancer Ther. 19, 1856–1865 (2020). 32669316 10.1158/1535-7163.MCT-20-0222 PMC7611352

[46] A Working Group of the NIH Office of AIDS Research Advisory Council (OARAC). Guidelines for the Prevention and Treatment of Opportunistic Infections in Adults and Adolescents With HIV (NIH, 2024).

[47] Hakki M Immune reconstitution to cytomegalovirus after allogeneic hematopoietic stem cell transplantation: impact of host factors, drug therapy, and subclinical reactivation. Blood 102, 3060–3067 (2003). 12843000 10.1182/blood-2002-11-3472

[48] Pastore D Recovery of CMV-specific CD8+ T cells and Tregs after allogeneic peripheral blood stem cell transplantation. Biol. Blood Marrow Transplant. 17, 550–557 (2011). 20457268 10.1016/j.bbmt.2010.04.011

[49] Ren J Generation and optimization of off-the-shelf immunotherapeutics targeting TCR-Vbeta2+ T cell malignancy. Nat. Commun. 15, 519 (2024). 38225288 10.1038/s41467-024-44786-2 PMC10789731

[50] Petersen J T-cell receptor recognition of HLA-DQ2-gliadin complexes associated with celiac disease. Nat. Struct. Mol. Biol. 21, 480–488 (2014). 24777060 10.1038/nsmb.2817

[51] Ting YT A molecular basis for the T cell response in HLA-DQ2.2 mediated celiac disease. Proc. Natl Acad. Sci. USA 117, 3063–3073 (2020). 31974305 10.1073/pnas.1914308117 PMC7022145

[52] May E Conserved TCR beta chain usage in reactive arthritis; evidence for selection by a putative HLA-B27-associated autoantigen. Tissue Antigens 60, 299–308 (2002). 12472659 10.1034/j.1399-0039.2002.600404.x

[53] Penkava F Single-cell sequencing reveals clonal expansions of pro-inflammatory synovial CD8 T cells expressing tissue-homing receptors in psoriatic arthritis. Nat. Commun. 11, 4767 (2020). 32958743 10.1038/s41467-020-18513-6 PMC7505844

[54] Komech EA TCR repertoire profiling revealed antigen-driven CD8+ T cell clonal groups shared in synovial fluid of patients with spondyloarthritis. Front. Immunol. 13, 973243 (2022). 36325356 10.3389/fimmu.2022.973243 PMC9618624

[55] Britanova OV Targeted depletion of TRBV9^+^ T cells as immunotherapy in a patient with ankylosing spondylitis. Nat. Med. 29, 2731–2736 (2023). 37872223 10.1038/s41591-023-02613-z PMC10667094

[56] Durham LE Linking skin and joint inflammation in psoriatic arthritis through shared CD8+ T cell clones. Arthritis Rheumatol. 10.1002/art.432862025 (2025). PMC12854012 40528683

[57] Direskeneli H Oligoclonal T cell expansions in patients with Behcet’s disease. Clin. Exp. Immunol. 117, 166–170 (1999). 10403931 10.1046/j.1365-2249.1999.00931.x PMC1905484

[58] Zou J Comprehensive analysis of T-cell receptor repertoires reveals antigen-driven T-cell clusters in patients with Behcet’s syndrome. Eur. J. Immunol. 53, e2250181 (2023). 36747316 10.1002/eji.202250181

[59] Xiong H Analysis of CD8^+^ TCRbeta chain repertoire in peripheral blood of vitiligo via high-throughput sequencing. Mol. Immunol. 160, 112–120 (2023). 37421821 10.1016/j.molimm.2023.06.009

[60] Alachkar H & Nakamura Y Deep-sequencing of the T-cell receptor repertoire in patients with haplo-cord and matched-donor transplants. Chimerism 6, 47–49 (2015). 26745665 10.1080/19381956.2015.1128624 PMC5154355

[61] Swindells MB abYsis: integrated antibody sequence and structure—management, analysis, and prediction. J. Mol. Biol. 429, 356–364 (2017). 27561707 10.1016/j.jmb.2016.08.019

[62] Kinde I, Wu J, Papadopoulos N, Kinzler KW & Vogelstein B Detection and quantification of rare mutations with massively parallel sequencing. Proc. Natl Acad. Sci. USA 108, 9530–9535 (2011). 21586637 10.1073/pnas.1105422108 PMC3111315

[63] Paul S T cell receptor signals to NF-kappaB are transmitted by a cytosolic p62-Bcl10-Malt1-IKK signalosome. Sci. Signal. 7, ra45 (2014). 24825920 10.1126/scisignal.2004882 PMC6450650

[64] Paul S, Kashyap AK, Jia W, He YW & Schaefer BC Selective autophagy of the adaptor protein Bcl10 modulates T cell receptor activation of NF-kappaB. Immunity 36, 947–958 (2012). 22658522 10.1016/j.immuni.2012.04.008 PMC3389288

[65] Paul S & Schaefer BC Visualizing TCR-induced POLKADOTS formation and NF-kappaB activation in the D10 T-cell clone and mouse primary effector T cells. Methods Mol. Biol. 1280, 219–238 (2015). 25736751 10.1007/978-1-4939-2422-6_12

[66] Traver MK, Paul S & Schaefer BC T cell receptor activation of NF-kappaB in effector T cells: visualizing signaling events within and beyond the cytoplasmic domain of the immunological synapse. Methods Mol. Biol. 1584, 101–127 (2017). 28255699 10.1007/978-1-4939-6881-7_8

