# Supplementary material for: TRBC2-targeting antibody–drug conjugates for the treatment of T cell cancers
Source: Nat Cancer. Author manuscript; Available in PMC 2026 Apr 11. (PMC13070007; doi:10.1038/s43018-025-01069-z)
Supplement: 1 [file NIHMS2134942-supplement-1.pdf]

nature cancer

Supplementary information https://doi.org/10.1038/s43018-025-01069-z

TRBC2-targeting antibody–drug conjugates for the treatment of T cell cancers

In the format provided by the authors and unedited

Table of contents

Supplementary Figure 1. Flow cytometry gating strategies.

Supplementary Figure 2. Flow cytometry gating strategies.

Supplementary Table 1. SPR measurements.

Supplementary Table 2. Cytotoxicity of anti-TRBC2 ADCs in T-cell cancer cell lines.

Supplementary Table 3. Anti-TRBC2 antibody sequences.

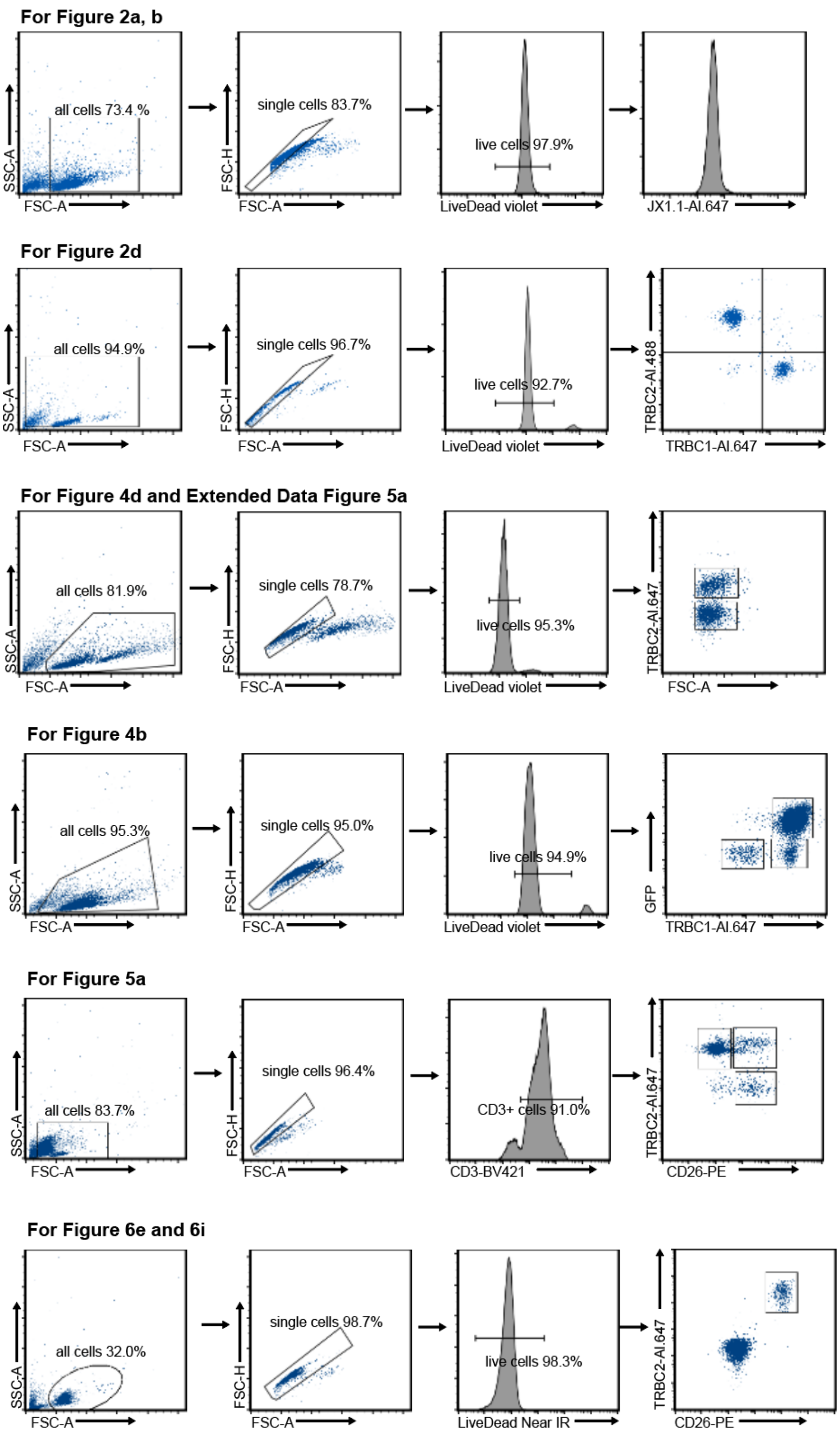

Supplementary Figure 1. Flow cytometry gating strategies. Representative flow cytometry figures and gating strategies of the indicated experiments in the main figures and extended data figures.

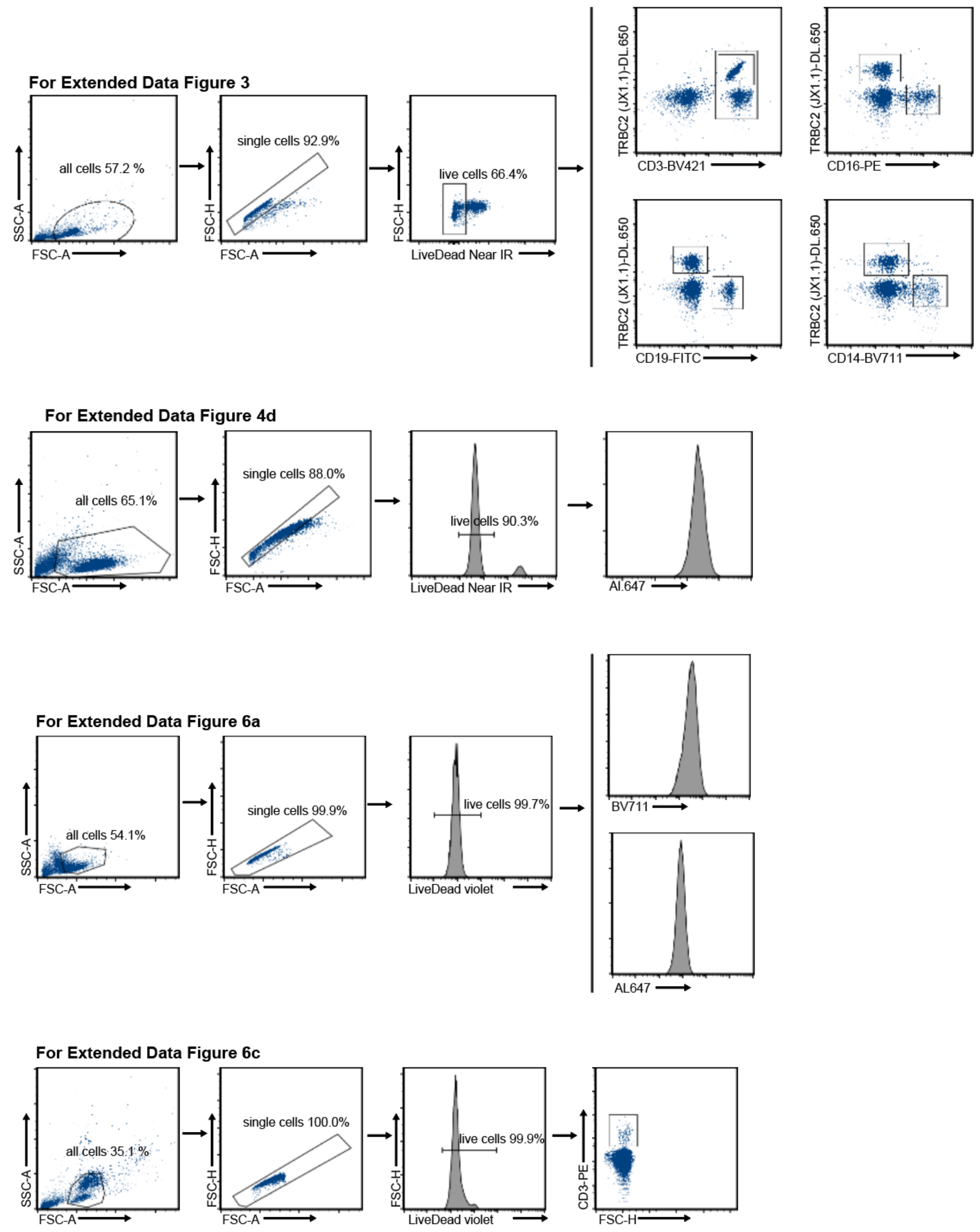

Supplementary Figure 2. Flow cytometry gating strategies. Representative flow cytometry figures and gating strategies of the indicated experiments in the main figures and extended data figures.

Antibody	TRBC2 protein			TRBC1 protein		
	k_a (1/Ms)	k_d (1/s)	K_D (nM)	k_a (1/Ms)	k_d (1/s)	K_D (nM)
**YR3-A5**	1.416×10^5	0.003198	23	NA	NA	NA
**KFN**	2.200×10^4	0.003852	175	NA	NA	NA
**KFNM**	5.222×10^4	0.003192	61	1.847×10^4	0.003189	173
**SAM.2**	5.979×10^4	0.003744	63	2.289×10^4	0.004574	200
**JX1.1**	1.287×10^5	0.001303	10	NA	NA	NA

Supplementary Table 1: SPR measurements. NA = not applicable. K_D values approximated to the nearest whole number. Data representative of one (KFNM) or two (YR3-A5) or three (KFN, SAM.2, JX1.1) independent experiments.

ADC	IC$_{50}$ (ng/mL)							
	TRBC2^{+} cell lines				TRBC2^{-} cell lines			
	HPB-ALL TRBC2^{+}	MHH-TALL2 TRBC2^{+}	HD-MAR-2 TRBC2^{+}	Jurkat TRBC2^{+}	HPB-ALL TCR-KO	H9 TRBC1^{+}	Jurkat TCR-KO	Jurkat TRBC1^{+}
**JX1.1-SG3249**	0.9	0.3	1.8	0.7	>1e^{5}	>1e^{5}	3127	>1e^{5}
**YR3-A5-SG3249**	12.5	11.1	133.7	3.0	270	2920	730	>1e^{5}
**SAM.2-SG3249**	22.8	8.0	217.8	43.9	1401	842	2137	>1e^{5}
**KFN-SG3249**	213	47.51	750.7	323.2	>1e^{5}	2399	>1e^{5}	5189

Supplementary Table 2: Cytotoxicity of anti-TRBC2 ADCs in T-cell cancer cell lines. IC$_{50}$ expressed in ng/mL. >1e^{5} indicates the estimated IC$_{50}$ values are above 10,000 ng/mL. Data from one experiment.

Antibody clone	V_L	V_H	Ref
**YR3-A5**	DVVMTQSPLSLPVTPGEPASISCRSS QNLVHSNGRTYLQWYLQKPGQSPQL LIYRVSNRFPGVPDRFSGSGSGTDFT LKISRVEAEDVGVYFCSQSTHVPYTF GGGTKVEIKR	QVQLVQSGAEVKKPGASVKVSCKASP RGFHGYHMHWVRQAPGQGLEWMGFI NPYNDDIQSNERFRGRVTMTSDKSTTT AYMELSSLRSEDTAVYYCARGNGKWG DGAYRFFDFWGQGTLVTVSS	Patent WO2022047 046A1
**JX1.1**	DVVMTQSPLSLPVTPGEPASISCRSS QNLKHRNGRTYLQWYLQKPGQSPQL LIYRVSNRFPGVPDRFSGSGSGTDFT LKISRVEAEDVGVYFCSQSTHVPYTF GGGTKVEIKR	QVQLVQSGAEVKKPGASVKVSCKASP YGFHGYHMHWVRQAPGQGLEWMGFI NPYNDDIQSNERFRGRVTMTSDKSTTT AYMELSSLRSEDTAVYYCARGNGKWG DGAYRFFDFWGQGTLVTVSS	This study
**KFN**	DIVMTQSPLSLPVTPGEPASISCRSSQ RLVHSNGNTYLHWYLQKPGQSPRLLI YRVSNRFPGVPDRFSGSGSGTDFTLK ISRVEAEDVGVYYCSQSTHVPYTFGQ GTKLEIKR	QVQLVQSGAEVKKPGASVKVSCKASG YKFTGFVMHWVRQAPGQGLEWMGFI NPYNDDIQSNERFRGRVTMTRDTSIST AYMELSRLRSDDTAVYYCARGNGYNF DGAYRFFDFWGQGTMVTVSS	Ref 17. Ferrari, M. et al. Nat Com 2024

Supplementary Table 3. Anti-TRBC2 antibody sequences. Ferrari M et al refers to Ferrari, M., Righi, M., Baldan, V. et al. Structure-guided engineering of immunotherapies targeting TRBC1 and TRBC2 in T cell malignancies. Nat Commun 15, 1583 (2024).
